# Ground reaction forces and muscle activity while walking on sand versus stable ground in individuals with pronated feet compared with healthy controls

**DOI:** 10.1371/journal.pone.0223219

**Published:** 2019-09-26

**Authors:** AmirAli Jafarnezhadgero, Amir Fatollahi, Nasrin Amirzadeh, Marefat Siahkouhian, Urs Granacher

**Affiliations:** 1 Department of Physical Education and Sport Sciences, Faculty of Educational Sciences and Psychology, University of Mohaghegh Ardabili, Ardabil, Iran; 2 Division of Training and Movement Sciences, Research Focus Cognition Sciences, University of Potsdam, Potsdam, Germany; University of Belgrade, SERBIA

## Abstract

**Background:**

Sand is an easy-to-access, cost-free resource that can be used to treat pronated feet (PF). Therefore, the aims of this study were to contrast the effects of walking on stable ground versus walking on sand on ground reaction forces (GRFs) and electromyographic (EMG) activity of selected lower limb muscles in PF individuals compared with healthy controls.

**Methods:**

Twenty-nine controls aged 22.2±2.5 years and 30 PF individuals aged 22.2±1.9 years were enrolled in this study. Participants walked at preferred speed and in randomized order over level ground and sand. A force plate was included in the walkway to collect GRFs. Muscle activities were recorded using EMG system.

**Results:**

No statistically significant between-group differences were found in preferred walking speed when walking on stable ground (PF: 1.33±0.12 m/s; controls: 1.35±0.14 m/s; p = 0.575; d = 0.15) and sand (PF: 1.19±0.11 m/s; controls: 1.23±0.18 m/s; p = 0.416; d = 0.27). Irrespective of the group, walking on sand (1.21±0.15 m/s) resulted in significantly lower gait speed compared with stable ground walking (1.34±0.13 m/s) (p<0.001; d = 0.93). Significant main effects of “surface” were found for peak posterior GRFs at heel contact, time to peak for peak lateral GRFs at heel contact, and peak anterior GRFs during push-off (p<0.044; d = 0.27–0.94). Pair-wise comparisons revealed significantly smaller peak posterior GRFs at heel contact (p = 0.005; d = 1.17), smaller peak anterior GRFs during push-off (p = 0.001; d = 1.14), and time to peak for peak lateral GRFs (p = 0.044; d = 0.28) when walking on sand. No significant main effects of “group” were observed for peak GRFs and their time to peak (p>0.05; d = 0.06–1.60). We could not find any significant group by surface interactions for peak GRFs and their time to peak. Significant main effects of “surface” were detected for anterior-posterior impulse and peak positive free moment amplitude (p<0.048; d = 0.54–0.71). Pair-wise comparisons revealed a significantly larger peak positive free moment amplitude (p = 0.010; d = 0.71) and a lower anterior-posterior impulse (p = 0.048; d = 0.38) when walking on sand. We observed significant main effects of “group” for the variable loading rate (p<0.030; d = 0.59). Pair-wise comparisons revealed significantly lower loading rates in PF compared with controls (p = 0.030; d = 0.61). Significant group by surface interactions were observed for the parameter peak positive free moment amplitude (p<0.030; d = 0.59). PF individuals exhibited a significantly lower peak positive free moment amplitude (p = 0.030, d = 0.41) when walking on sand. With regards to EMG, no significant main effects of “surface”, main effects of “group”, and group by surface interactions were observed for the recorded muscles during the loading and push-off phases (p>0.05; d = 0.00–0.53).

**Conclusions:**

The observed lower velocities during walking on sand compared with stable ground were accompanied by lower peak positive free moments during the push-off phase and loading rates during the loading phase. Our findings of similar lower limb muscle activities during walking on sand compared with stable ground in PF together with lower free moment amplitudes, vertical loading rates, and lower walking velocities on sand may indicate more relative muscle activity on sand compared with stable ground. This needs to be verified in future studies.

## Introduction

Pronated feet (PF) are characterized by a lowered medial longitudinal arch during the weight-bearing phase that resolves during non-weight bearing. PF prevalence rates range from 48% to 78% in youth aged 2–16 years [1] and 2–23% in adults [2]. Individuals with symptomatic PF walk at lower preferred speed [3] and lower walking cadence [4], and are significantly more likely to suffer from hip, knee, and back pain [3, 5]. There is evidence that altered foot architecture affects lower limbs alignment [4, 6] which again appears to have an impact on erector spinae and gluteal muscle activities [7].

When it comes to kinetics or plantar pressure distribution during walking, it has been shown that PF individuals compared with healthy controls exhibit lower second peak vertical ground reaction forces (GRFs) [8], lower peak pressure and maximal force in the lateral forefoot, higher maximal force in the medial midfoot [9], higher anterior posterior impulses [10], and higher invertor moments [11]. With regards to kinematics, over PF is associated with an internal rotation of the shank [12], and pelvic ipsilateral drop during weight-bearing when walking [13].

For muscle activity, there is evidence that PF individuals demonstrate higher activities of selected muscles encompassing the ankle joint (i.e., tibialis posterior and anterior, toe flexors, plantar flexors) and lower activities of particularly the evertor muscles (i.e., peroneus muscle) [11, 14, 15].

Given the above reported changes in kinetics, kinematics and lower limbs muscle activities in PF individuals compared with healthy controls, specific passive (i.e., orthotics) and/or active (e.g., strength training) treatment types are needed. Of note, PF may develop from deficits in muscle strength and stability or from overuse [16–18]. It has previously been postulated that the triceps surae, peronei, tibialis posterior, and anterior muscles act as dynamic stabilizers of the medial longitudinal arch [19–21]. There is evidence that fatigue of the intrinsic foot muscles results in increased foot pronation as indicated by navicular drop [19]. Similar results were found following the injection of lidocaine to knock out activity of the tibial nerve to the medial malleolus [22]. These studies clearly indicate that the foot muscles play a vital role in stabilizing the medial longitudinal arch [11, 23].

Surprisingly, the effects of active types of treatment (i.e., exercise) on pain and function of individuals with over PF have hardly been investigated in the literature. A recent study examined the combined effects of orthoses, stretching, and strength training on pain and function [24]. After a three months intervention period, less pain and a longer distance covered in the 5-minute walk test were noted [24].

Sand is a promising candidate to be included during therapy because it is cost-free, and many people around the globe have access to it. More importantly, sand is an unstable and unpredictable surface that could positively affect the biomechanics of human locomotion. A previous study has demonstrated that people with multiple sclerosis adapted to walking on sand by significantly increasing hip and knee flexion and ankle dorsiflexion during the swing phase [25]. To the authors’ knowledge, there is no study available that examined the effects of walking on sand versus stable ground in individuals with PF compared with healthy controls. Therefore, the aim of this study was to contrast the effects of walking at preferred speed on sand versus stable ground on GRF and activities of selected lower limb muscles in individuals with PF compared with healthy age-matched controls. With reference to the relevant literature [25], we hypothesized slower walking speed together with lower loading rates, and higher free moment (FM) and muscle activities when walking on sand compared with stable ground in both experimental groups. We further expected that these results would particularly be prevalent in PF individuals [10, 17].

## Materials and methods

### Participants

We used the freeware tool G*Power (http://www.gpower.hhu.de/) to calculate a one-sided a priori power analysis with the F test family (ANOVA repeated measures within-between interaction) and the respective statistical test based on a related study that examined walking kinetics in adults with PF [26]. The power analysis was computed with an assumed Type I error of 0.05, a Type II error rate of 0.20 (80% statistical power), 2 tests (pre, post), a correlation coefficient of 0.5 between observations, and an effect size of 0.80 (i.e., interaction effects) for walking kinetics (i.e., peak vertical GRF). The analysis revealed that 30 participants would be sufficient to observe large Group x Time interactions. According to Cohen, a large effect size (>0.8) implies that the means of the two experimental groups differ by 0.8 standard deviations [27]. Participants (age range: 18–26 year) were recruited in physical therapy clinics in Ardabil city, Iran in January 2019. Twenty-nine healthy individuals (12 females, 17 males) and 30 individuals with over PF (15 females, 15 males) were eligible to participate in this study (Table 1).

**Table 1:** Participants’ characteristic.

Variables	Group with over pronated feet	Healthy controls	Between-group differences (p-values)
Age (year)	22.2 ± 1.9	22.2 ± 2.5	0.985
Body height (cm)	169.3 ± 5.5	178.0± 6.6	0.103
Body mass (kg)	68.4 ± 8.4	75.0 ± 8.2	0.198
Body mass index (kg/m^2^)	25.2 ± 5.3	23.8 ± 3.4	0.372
Navicular drop (mm)	12.4 ± 1.7	5.2 ± 0.9	<0.001
Foot posture index	11.0 ±0.5	3.4 ± 0.4	<0.001

All participants were right footed and right handed as determined by a kicking and throwing ball test. An orthopedic surgeon from a local clinic assessed all participants prior to the start of the study. An individual was included in the healthy control group if he or she did not have any signs of musculoskeletal, postural, or neurological disorders. For the over PF group, participants were recruited if they showed a navicular drop of more than 10 mm [17], and a foot posture index larger than 10 [17]. Of note, the navicular drop was measured as the difference in navicular height during non-weight bearing compared with full weight bearing of the foot during quiet unilateral standing [28]. For both experimental groups, a priori defined exclusion criteria comprised a history of musculoskeletal surgery at the trunk and/or lower limbs, neuromuscular or orthopedic disorders (except of over PF for over PF group), limb length differences larger than 5 mm, and the performance of strenuous physical exercises ≤ 2 days prior to testing. The research protocol was approved by the ethics committee of the Medical Sciences University of Ardabil, Iran (IR.ARUMS.REC.1398.119). All participants provided their written informed consent to participate in this study.

### Experimental set-up and data processing

**Fig 1:**
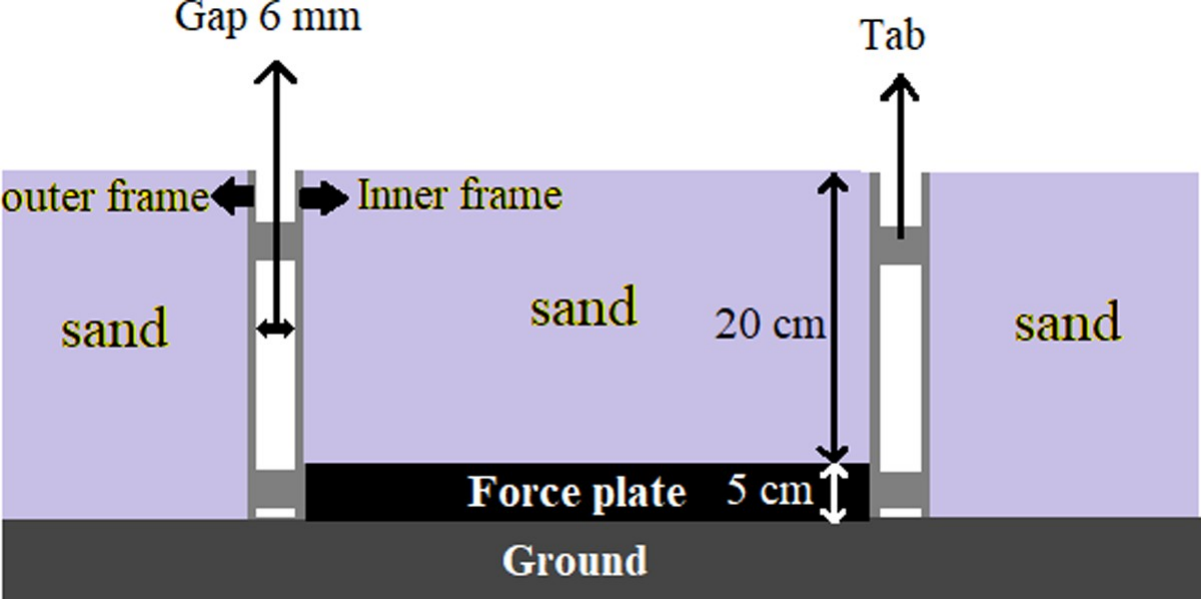
Schematic figure of the frame around the walkway.

Two adjustable walkways (10 m long, 1 m wide and 0.25 m deep) with a specifically constructed frame were built to conduct this study. One walkway was covered with reinforced plywood and an embedded force plate (Bertec Corporation, Columbus, OH, USA) to replicate a stable surface environment. The unstable walkway was filled with sand to simulate a common deformable surface environment. A Bertec force plate was embedded in the walkway (20 cm underneath the sand) and used to collect GRF data at 1000 Hz. The frame consisted of two welded steel rectangles concentrically aligned with 6 mm clearance between the walls. The outer frame was securely attached to the base of each walkway. The inner frame was attached to the force plate using four alignment tabs (Fig 1). The alignment tabs ensured that all shear forces were transmitted to the surface of the force plate. Previous studies confirmed that this force plate construction embedded into the walkway is well-suited to reduce dissipation of force and to accurately identify forces while walking on the two surface conditions [29–32]. Test-retest reliability was assessed for GRF and EMG data and revealed intra-class correlation coefficients (ICC) > 0.6 and 0.53, respectively.

Kinetic data were processed as described by Jafarnezhadgero et al. [33]. GRFs were low-pass filtered at 20 Hz (4th order Butterworth filter, zero lag). Specific gait characteristics (heel strike and toe off) were identified using the Bertec force plate. For this purpose, a 10 N threshold was used to detect the stance phase of the gait cycle. The following dependent variables were extracted from GRF data [33]: First (Fz_HC_) and second vertical peak force (Fz_PO_) as well as the minimum force between peaks (Fz_MS_). Braking (Fy_HC_) and propulsion forces (Fy_PO_) were recorded from the anterior-posterior force curve. From the medial-lateral curve, we calculated the positive (lateral) peak (Fx_HC_) which occurs right after heel contact. Moreover, we additionally assessed the negative peak which corresponds to the transfer of body mass to the supporting limb (Fx_MS_) and subsequently to the contralateral limb (Fx_PO_). GRF amplitudes were normalized to body weight (BW) and reported in %BW. Time to peak (TTP) was defined as the time between the initial heel contact and the corresponding peak of GRF components. Loading rate was defined as the slope between heel contact and Fz_HC_ on the vertical force curve. Impulse was calculated using the trapezoidal integration method and expressed as follows:

$$Impulse=\Delta t\left(\left(\frac{F_{1}+F_{n}}{2}\right)+\;\overset{n-1}{\underset{i=2}{\sum }}Fi\right)$$

In this equation, delta t is the time period for which the impulse was calculated, F_1_ and F_n_ are reaction forces at the first and the last frame. The FM of the foot was computed as follows:

$$FM=M_{z}+\left(F_{x}\times COP_{y}\right)-\left(F_{y}\times COP_{x}\right)$$

Where, Mz is the moment around the vertical axis; x and y are the horizontal components of the center of pressure (COP), and Fx, Fy are the horizontal GRF components. Moreover, FM amplitudes were normalized with regards to BW × height. All gait variables were averaged across three trials [33]. For stance phase analysis, GRF and COP data were normalized to 101 data points. For COP analysis, deformation height was computed. Of note, deformation height corresponds to the maximum vertical slippage depth while walking on sand [34]. The COP values were calculated in accordance with Xu et al. [34].

A wireless EMG system (EMG Pre-Amplifier, Biometrics Ltd, Nine Mile Point Ind. Est, Newport, UK) with eight pairs of bipolar Ag/AgCl surface electrodes (25 mm center-to-center distance; input impedance of 100 MΩ; and common mode rejection ratio of >110 dB) was used to record the activity of the tibialis anterior (TA), gastrocnemius medialis (Gas-M), biceps femoris (BF), semitendinosus (ST), vastus lateralis (VL), vastus medialis (VM), and rectus femoris (RF), and gluteus medius (Glut-M) muscles of the right leg [35]. A die cut medical grade double-sided adhesive tape (T350, Biometrics Ltd, Nine Mile Point Ind. Est, Newport, UK) was used to attach the electrodes to the muscle bellies. The raw EMG signals were digitized at 1000 Hz and streamed via Bluetooth to a computer for further analysis. According to the European recommendations for surface EMG (SENIAM), skin surface was shaved and cleaned with alcohol (70% Ethanol–C2H5OH) over the selected muscles. Thereafter, the skin was abraded gently prior to electrode placement [35]. GRF and EMG data were synchronized using Nexus software (Oxford Metrics, Oxford, UK). For EMG analyses, the gait cycle was divided into the following phases: loading phase (0–20% of gait cycle), mid-stance (20–47% of gait cycle), push off (47–70% of gait cycle), and swing phase (70–100% of gait cycle) [36]. Using a handheld dynamometer, maximum voluntary isometric contraction (MVIC) was assessed for each recorded muscle to normalize EMG during walking to maximal voluntary activation. Table 2 describes muscle specific MVIC tests.

**Table 2:** Description of the maximum voluntary isometric contraction (MVIC) tests for tibialis anterior (TA), gastrocnemius medialis (Gas-M), biceps femoris (BF), semitendinosus (ST), vastus lateralis (VL), vastus medialis (VM), rectus femoris (RF), and gluteus medius (Glut-M) muscles.

Muscles	Test protocol
TA	In seated position on a chair with back rest, with 90° hip knee, and ankle joint flexion. Participants were asked to activate TA at maximal effort against resistance [17].
Gas-M	In seated position on the examination table with the hip flexed by 90° and the knee and ankle in neutral position. Participants activated their plantar flexors at maximal effort against resistance [17].
BF	In seated position on a chair with hip and knees flexed at 90°. Participants activated the hamstring muscles at maximal effort against resistance [17].
ST	In seating position on a chair with hip and knees flexed at 90°. Participants maximally activated their knee flexors against resistance [17].
VL	In seated position on a chair with hip and knees flexed at 90°. Participant maximally activated their knee extensors against resistance [17].
VM	In seated position on a chair with hip and knees flexed at 90°. Participants maximally activated their knee extensors against resistance [17].
RF	In seated position on a chair with hip and knees flexed at 90°. Participants maximally activated their knee extensors against resistance [17].
Glut-M	In standing position, participants maximally activated their hip abductors against resistance.

### Experimental procedures

All participants wore the same shoe model according to their foot size. Prior to testing, participants conducted a standardized 5 min warm-up protocol consisting of 2 min jogging at low-to-moderate intensity followed by 3 minutes of stretching. For the walking trials, participants were familiarized with the laboratory situation and walked across each walkway three times. During familiarization, the optimal distance to the force plate was identified to increase the likelihood of force plate contact during the walkway trials. During testing, participants had to conduct at least eight steps before they hit the force plate with their right foot. Participants walked at preferred speed and in randomized order over level (stable) ground and sand. A trial was considered successful if the foot landed in the middle of the force plate and if EMG signals were artefact free upon visual examination of the online screen. After the walking trials, MVIC exercises for each muscle were taken to normalize EMG (Table 2). Three successful walking trials were assessed for each condition and used for further data analysis.

### Statistical analyses

Data are presented as group mean values and standard deviations. After normal distribution was examined and confirmed using the Shapiro-Wilk-Test, a separate 2 (surface: stable ground vs sand) × 2 (groups: healthy vs PF) ANOVA with repeated measures was computed. Post hoc analyses were calculated using Bonferroni adjusted paired sample t-tests. Additionally, effect sizes were determined by converting partial eta-squared (η^2^_p_) to Cohen’s d. According to Cohen [27], d< 0.50 indicate small effects, 0.50≤d< 0.80 indicate medium effects, and d≥0.80 indicate large effects. The significance level was set at p< 0.05. All analyses were performed using Statistical Package for Social Sciences (SPSS) version 24.0.

## Results

No statistically significant between group differences (PF vs healthy controls) were found for all anthropometric measures as well as for preferred gait speed when walking on stable ground (over PF group: 1.33±0.12 m/s, healthy controls: 1.35±0.14 m/s; p = 0.575; d = 0.15) and sand (over PF group: 1.19±0.11 m/s, healthy controls: 1.23±0.18 m/s; p = 0.416; d = 0.27). Irrespective of the experimental group, walking on sand (1.21±0.15 m/s) resulted in significantly lower gait speed compared with stable ground walking (1.34±0.13 m/s) (p<0.001; d = 0.93). In the PF group, walking on sand (1.19±0.11 m/s) resulted in a significantly slower self-selected gait speed compared with stable ground (1.32±0.12 m/s) (p<0.05; d = 1.13). Similar changes in gait speed were noticed in the healthy control group when walking on sand (1.22±0.18 m/s) compared with stable ground (1.34±0.14 m/s) (p<0.05; d = 0.75). The statistical analyses indicated significant main effects of “surface” for stance time during walking (p<0.034; d = 0.58). Pair-wise comparisons revealed a significantly longer stance time when walking on sand (0.78±0.17 ms) compared with walking on stable ground (0.74±0.01 ms) (p = 0.034, d = 0.44). No statistically significant main effects of “group” were detected for stance time during walking (p>0.05; d = 0.28). Moreover, the statistical analysis did not yield any significant group by surface interactions for stance time during walking (p>0.05; d = 0.24).

Significant main effects of “surface” were found for Fy_HC,_ Fy_PO,_ and TTP Fx_HC_ (p<0.044; d = 0.27–0.94) (Table 3). Pair-wise comparisons revealed significantly smaller Fy_HC_ (p = 0.005; d = 1.17), Fy_PO_ (p = 0.001; d = 1.14), and TTP Fx_HC_ (p = 0.044; d = 0.28) when walking on sand compared with stable ground (Table 3). Furthermore, we could not detect any significant main effects of “group” for peak GRFs and their TTP (p>0.05; d = 0.06–1.60) (Table 3).

**Table 3:** Data are means and standard deviations for ground reaction forces (GRF) during walking on sand versus stable ground in individuals with over pronated feet (PF) compared with healthy controls.

GRF	Over PF group				Healthy controls				Sig. (Effect size)		
	Stable ground	Sand	95% CI	%Δ	Stable ground	Sand	95% CI	%Δ	Surface	Group	Surface x Group
Fz_HC_	110.51±18.85	108.45±15.19	-1.3,8.1	-2.57	107.11±16.15	103.53±7.06	-2.7,5.5	-3.34	0.192(0.35)	0.475(0.19)	0.851(0.06)
Fz_MS_	78.56±11.36	80.13±6.50	-4.8,1.6	1.99	79.52±7.43	77.33±9.87	-1.4,5.7	-2.75	0.427(0.21)	0.223(0.33)	0.301(0.28)
Fz_PO_	109.36±16.27	107.78±14.60	-0.7,3.9	-1.44	107.39±5.86	105.35±7.51	-0.2,4.2	-1.89	0.103(0.44)	0.852(0.06)	0.490(0.19)
Fx_HC_	7.98±3.11	7.79±3.16	-1.5,1.9	-2.38	7.45±2.92	7.03±3.85	-0.8,1.7	-5.63	0.107(0.44)	0.632(0.13)	0.543(0.17)
Fx_PO_	-5.40±3.13	-7.07±4.79	-0.3,3.6	30.92	-6.75±3.88	-9.05±5.07	0.6,3.9	34.07	0.964(0.00)	0.231(0.33)	0.952(0.00)
Fy_HC_	-7.80±6.73	-4.65±5.40	-6.7,-1.9	-40.38	-4.65±5.40	-3.62±1.33	-3.0,1.0	-22.15	**0.005(0.78)**	0.463(0.20)	0.252(0.31)
Fy_PO_	8.46±5.51	4.62±2.31	1.6,6.0	-45.39	5.84±5.32	4.06±1.44	-0.3,3.9	-30.47	**0.001(0.94)**	0.456(0.20)	0.810(0.06)
TTP Fz_HC_	170.50±66.06	174.63±71.02	-27.1,18.8	2.42	174±58.94	196.31±122.73	-71.5,26.9	12.82	0.965(0.00)	0.433(0.21)	0.640(0.13)
TTP Fz_MS_	329.26±52.13	353.10±62.59	-54.5,6.9	7.24	341.65±63.06	370.48±171.70	-92.3,34.73	8.43	0.619(0.13)	0.799(0.06)	0.770(0.09)
TTP Fz_PO_	536.00±63.39	550.43±46.59	-39.3,10.4	2.69	538.58±74.38	554.89±179.43	-87.8,55.1	3.02	0.773(0.06)	0.526(0.17)	0.949(0.00)
TTP Fx_HC_	22.26±11.47	25.58±19.06	-10.4,3.8	14.91	19.14±16.75	12.36±11.43	-1.2,14.7	-35.42	**0.044(0.27)**	0.386(0.23)	0.420(0.22)
TTP Fx_PO_	442.76±162.98	483.63±160.56	-116.0,34.3	9.23	384.10±193.50	432.17±195.62	-121.2,25.0	12.51	0.532(0.17)	0.115(0.43)	0.859(0.06)
TTP Fy_HC_	128.46±57.95	126.43±52.61	-21.5,25.6	-1.58	151.75±66.33	160.68±122.19	-61.9,44.0	5.88	0.130(0.41)	0.445(0.20)	0.318(0.27)
TTP Fy_PO_	608.46±66.07	588.93±114.50	-34.3,73.3	-3.20	591.62±115.30	546.17±165.36	-34.1,124.9	-7.68	0.908(0.00)	0.158(1.60)	0.714(0.09)

Legends: Fz_HC,_ peak vertical ground reaction force during heel contact; Fz_MS_, vertical ground reaction force during mid stance; Fz_PO,_ peak vertical ground reaction force during push of phase; Fy_HC_, braking force; Fy_PO_, propulsion force; Fx_HC_, peak lateral ground reaction force during heel contact; Fx_HC_, peak medial ground reaction force during push of phase; TTP, time to peak; CI, confidence interval.

Finally, no significant group by surface interactions were found for peak GRFs and their TTP) p>0.05; d = 0.00–0.31) (Table 3).

The statistical analyses indicated significant main effects of “surface” for impulse y and peak positive FM amplitude (p<0.048; d = 0.54–0.71) (Table 4). Pair-wise comparisons revealed a significantly larger peak positive FM amplitude (p = 0.010; d = 0.71) and a lower impulse y (p = 0.048; d = 0.38) when walking on sand compared with walking on stable ground (Table 4).

Moreover, we observed significant main effects of “group” for the variable loading rate (p<0.030; d = 0.59) (Table 4). Pair-wise comparisons revealed significantly lower loading rates in the over PF group compared with the healthy controls (p = 0.030; d = 0.61) (Table 4).

**Table 4:** Data are means and standard deviations for impulses, free moments and vertical loading rate during walking on sand versus stable ground in individuals with over pronated feet (PF) compared with healthy controls.

Variables	Over PF group				Healthy controls				Sig. (Effect size)		
	Stable ground	Sand	95% CI	%Δ	Stable ground	Sand	95% CI	%Δ	Surface	Group	Surface x Group
Impulse x	3.18±2.22	4.12±3.29	-2.4,0.57	29.55	3.52±1.85	5.77±3.17	-3.4,-1.0	63.92	0.778(0.06)	0.252(0.31)	0.490(0.19)
Impulse y	3.45±3.81	2.33±3.24	-0.7,2.9	-32.46	2.25±1.26	1.96±1.62	-0.4,1.0	-12.88	**0.048(0.54)**	0.500(0.18)	0.939(0.00)
Impulse z	58.08±6.67	79.44±105.08	-59.9,17.2	36.77	57.86±8.71	61.21±16.66	-9.4,2.7	5.78	0.431(0.21)	0.486(0.19)	0.520(0.17)
Free Moment (negative) ×10^−3^	-0.88±0.62	-0.90±0.68	-0.3,0.5	2.27	-0.74±0.97	-1.06±0.98	-0.5,0.1	43.24	0.142(0.40)	0.222(0.33)	0.121(0.42)
Free Moment (positive) ×10^−3^	2.33±1.46	2.22±1.12	-0.3,0.3	-4.72	2.36±0.99	2.56±0.95	-.1,0.7	8.47	**0.010(0.71)**	0.167(0.37)	**0.030(0.59)**
Loading rate	8.06±4.82	7.64±4.92	-1.2,2.0	-5.21	9.33±17.33	9.93±16.65	-9.9,8.7	6.43	0.929(0.00)	**0.030(0.59)**	0.815(0.06)

Legends: x, medio-lateral direction; y, anterior-posterior direction; z, vertical direction; CI, confidence interval.

Finally, the statistical analysis showed significant group by surface interactions for the parameter peak positive FM amplitude (p<0.030; d = 0.59) (Table 4). Individuals with PF compared with healthy controls exhibited a significantly lower peak positive FM amplitude (p = 0.030, d = 0.41) when walking on sand compared with stable ground.

With regards to EMG activity, no statistically significant main effects of “surface” were found for activities of selected lower limb muscles during the loading phase) p>0.05; d = 0.00–0.48) (Table 5).

**Table 5:** Data are means and standard deviations for muscle activity during the loading phase (% maximum voluntary isometric contraction [MVIC]) while walking on stable ground and sand.

Muscles	Over PF group				Healthy controls				Sig. (Effect size)		
	Stable ground	Sand	95% CI	%Δ	Stable ground	sand	95% CI	%Δ	Surface	Group	Surface x Group
TA	23.06±12.37	24.90±11.76	-6.4,2.7	7.97	24.94±13.76	25.22±16.08	-5.2,4.6	1.12	0.728(0.09)	0.274(0.29)	0.724(0.09)
Gas-M	12.11±14.31	7.72±8.64	0.4,8.3	-36.25	12.66±11.20	11.86±12.15	-3.4,5.0	-6.31	0.346(0.25)	0.204(0.35)	0.341(0.25)
VL	21.26±14.53	21.06±16.51	-4.7,5.1	-0.94	17.28±15.19	16.34±14.25	-4.7,6.5	-5.43	0.170(0.37)	0.115(0.43)	0.450(0.20)
VM	20.78±13.52	16.36±12.46	-0.6,9.4	-21.27	22.36±17.67	27.58±21.46	-14.6,4.1	23.34	0.639(0.13)	0.645(0.13)	0.152(0.39)
RF	17.65±9.91	16.93±6.95	-1.9,3.3	-4.07	20.27±14.49	20.74±14.26	-2.1,1.1	2.31	0.610(0.14)	0.873(0.00)	0.349(0.25)
BF	9.26±5.02	10.95±9.47	-4.2,0.8	18.25	13.48±7.32	15.55±10.06	-6.4,2.2	15.35	0.974(0.00)	0.083(0.47)	0.938(0.00)
ST	15.00±20.85	11.05±9.90	-1.1,9.0	-26.33	14.66±10.83	14.44±14.65	-6.9,7.3	-1.50	0.481(0.19)	0.489(0.19)	0.550(0.15)
Glut-M	19.45±15.29	15.68±9.62	-2.3,9.9	-19.38	25.14±22.36	21.38±13.10	-5.0,12.5	-14.95	0.976(0.00)	0.410(0.22)	0.860(0.06)

Legends: PF, pronated feet; TA, tibialis anterior; Gas-M, gastrocnemius medialis; BF, biceps femoris; ST, semitendinosus; VL, vastus lateralis; VM, vastus medialis; RF, rectus femoris; Glut-M, gluteus medius; CI, confidence interval.

The statistical analyses did not demonstrate any significant main effects of “group” for activities of selected lower limb muscles during the loading phase) p>0.05; d = 0.00–0.47) (Table 5).

Finally, we did not find any significant group by surface interactions for activities of selected lower limb muscles during the loading phase) p>0.05; d = 0.00–0.39) (Table 5).

Of note, statistically significant main effects of “surface” were found for Gas-M activity during the mid-stance phase (p<0.003; d = 0.84) (Table 6). Pair-wise comparisons revealed significantly higher Gas-M activity (p = 0.003; d = 0.13) when walking on sand compared with walking on stable ground (Table 6).

**Table 6:** Data are means and standard deviations for muscle activity during the mid-stance phase (% maximum voluntary isometric contraction [MVIC]) while walking on stable ground and sand.

Muscles	Over PF group				Healthy controls				Sig. (Effect size)		
	Stable ground	Sand	95% CI	%Δ	Stable ground	Sand	95% CI	%Δ	Surface	Group	Surface x Group
TA	8.64±7.28	10.32±11.16	-5.0,1.7	19.44	10.74±11.31	12.85±11.95	-5.8,1.5	19.64	0.646(0.13)	0.243(0.31)	0.804(0.06)
Gas-M	30.26±22.44	32.50±32.13	-14.2,9.8	7.40	29.86±23.73	32.16±27.18	-12.1,7.5	7.70	**0.003(0.84)**	0.050(0.54)	0.146(0.39)
VL	11.92±13.19	14.03±17.83	-8.6,4.4	17.70	7.57±6.02	13.15±19.24	-12.1,1.0	73.71	0.124(0.42)	0.120(0.42)	0.832(0.06)
VM	12.22±12.64	11.88±13.13	-5.4,6.1	-2.78	18.86±22.63	21.43±17.05	-12.2,7.0	13.62	0.227(0.29)	0.149(0.39)	0.363(0.25)
RF	16.82±9.30	18.71±11.66	-6.0,2.2	11.23	20.45±15.35	20.73±15.66	-2.6,2.1	1.36	0.495(0.18)	0.825(0.06)	0.670(0.11)
BF	6.54±7.04	7.77±8.66	-3.4,1.0	18.80	8.62±6.57	12.82±15.02	-9.4,1.0	48.72	0.993(0.00)	0.564(0.15)	0.484(0.19)
ST	12.32±19.06	9.97±11.11	-4.4,9.1	-19.07	7.38±6.21	7.12±6.27	-3.1,3.6	-3.52	0.814(0.06)	0.211(0.34)	0.488(0.19)
Glut-M	15.76±15.12	15.11±10.96	-5.4,6.7	-4.12	19.32±16.83	21.53±20.19	-7.2,2.7	11.43	0.209(0.34)	0.377(0.24)	0.956(0.00)

Legends: PF, pronated feet; TA, tibialis anterior; Gas-M, gastrocnemius medialis; BF, biceps femoris; ST, semitendinosus; VL; vastus lateralis; VM, vastus medialis; RF, rectus femoris; Glut-M, gluteus medius; CI, confidence interval.

No statistically significant main effects of “group” were found for activities of selected lower limb muscles during the mid-stance phase) p>0.05; d = 0.06–0.54) (Table 6).

Moreover, no significant group by surface interactions were observed for activities of selected lower limb muscles during the mid-stance phase) p>0.05; d = 0.00–0.39) (Table 6).

No statistically significant main effects of “surface” were found for activities of selected lower limb muscles during the push off phase) p>0.05; d = 0.00–0.42) (Table 7).

**Table 7:** Data are means and standard deviations for muscle activity during the push off phase (% maximum voluntary isometric contraction [MVIC]) while walking on stable ground and sand.

Muscles	Over PF group				Healthy controls				Sig. (Effect size)		
	Stable ground	Sand	95% CI	%Δ	Stable ground	Sand	95% CI	%Δ	Surface	Group	Surface x Group
TA	9.01±6.55	8.86±6.07	-1.9,2.2	-1.66	11.33±10.19	12.91±13.99	-5.5,2.3	13.94	0.209(0.34)	0.652(0.13)	0.946(0.00)
Gas-M	46.95±23.74	51.07±23.05	-11.5,3.2	8.77	37.74±22.44	36.23±19.87	-8.4,11.5	-4.00	0.319(0.27)	0.344(0.25)	0.652(0.13)
VL	8.85±12.56	10.19±15.87	-5.5,2.8	15.14	7.17±9.15	10.04±12.32	-6.8,1.0	40.02	0.125(0.42)	0.054(0.53)	0.694(0.11)
VM	14.64±16.20	17.40±22.01	-9.8,4.2	18.85	18.39±19.19	18.07±16.01	-8.6,9.2	-1.74	0.318(0.27)	0.416(0.22)	0.911(0.00)
RF	17.83±11.98	15.61±7.49	-0.7,5.1	-12.45	20.59±14.34	21.24±15.73	-3.1,1.8	3.15	0.726(0.22)	0.963(0.00)	0.191(0.35)
BF	5.83±8.83	4.28±3.07	-1.8,4.9	-26.58	7.35±7.37	7.96±9.61	-4.1,2.9	8.29	0.900(0.00)	0.756(0.09)	0.365(0.25)
ST	7.87±11.74	4.99±5.13	-1.6,7.3	-36.59	8.28±14.40	7.35±6.93	-4.9,6.7	-11.23	0.829(0.06)	0.883(0.00)	0.598(0.14)
Glut-M	12.13±8.71	8.30±6.22	0.7,6.9	-31.57	16.85±17.94	19.88±14.97	-11.5,5.5	17.98	0.527(0.17)	0.152(0.39)	0.254(0.31)

Legends: PF, pronated feet; TA, tibialis anterior; Gas-M, gastrocnemius medialis; BF, biceps femoris; ST, semitendinosus; VL, vastus lateralis; VM, vastus medialis; RF, rectus femoris; Glut-M, gluteus medius; CI, confidence interval.

The statistical analyses did not demonstrate any significant main effects of “group” for activities of selected lower limb muscles during the push off phase) p>0.05; d = 0.00–0.53) (Table 7).

Moreover, we could not detect any significant group by surface interactions for activities of selected lower limb muscles during the push off phase) p>0.05; d = 0.00–0.35) (Table 7).

Of note, we observed significant main effects of “surface” for VM activity during the swing phase of walking (p<0.025; d = 0.61) (Table 8). Pair-wise comparisons revealed a significantly lower VM activity (p = 0.025; d = 0.69) when walking on sand compared with walking on stable ground (Table 8).

**Table 8:** Data are means and standard deviations for muscle activity during the swing phase (% maximum voluntary isometric contraction [MVIC]) while walking on stable ground and sand.

Muscles	Over PF group				Healthy controls				Sig. (Effect size)		
	Stable ground	Sand	95% CI	%Δ	Stable ground	Sand	95% CI	%Δ	Surface	Group	Surface x Group
TA	14.74±6.59	15.36±7.42	-2.8,1.5	-4.20	20.31±12.63	19.92±14.34	-2.7,3.4	-1.92	0.758(0.09)	0.868(0.00)	0.712(0.09)
Gas-M	12.93±13.72	11.37±21.08	-3.8,6.9	-12.06	16.22±16.18	15.33±16.24	-7.0,8.8	-5.48	0.893(0.00)	0.404(0.22)	0.799(0.06)
VL	12.80±21.43	9.34±16.84	0.1,6.8	-27.03	10.47±14.97	7.32±8.40	-1.1,7.4	-30.08	0.592(0.14)	**0.037(0.57)**	0.504(0.18)
VM	13.37±17.71	11.32±15.11	-0.7,4.8	-15.33	18.39±19.07	18.15±18.32	-8.6,9.1	-1.30	**0.025(0.61)**	0.842(0.06)	0.146(0.39)
RF	17.77±13.39	15.34±7.19	-0.6,5.5	-13.68	20.48±16.32	19.94±14.24	-1.2,2.3	-2.63	0.081(0.47)	0.706(0.11)	**0.045(0.55)**
BF	7.65±9.96	6.07±4.32	-1.0,4.1	-20.65	8.41±6.20	7.76±5.49	-1.6,2.9	-7.72	0.827(0.06)	0.967(0.00)	0.447(0.20)
ST	8.43±14.70	5.55±5.73	-1.0,6.8	-34.16	8.53±6.51	10.49±7.74	-5.5,1.6	22.97	0.505(0.18)	0.344(0.25)	0.162(0.38)
Glut-M	16.73±18.13	11.62±9.79	-1.5,11.7	-30.54	18.17±13.78	19.97±17.47	-8.3,4.7	9.90	0.241(0.31)	0.822(0.06)	0.353(0.25)

Legends: PF, pronated feet; TA, tibialis anterior; Gas-M, gastrocnemius medialis; BF, biceps femoris; ST, semitendinosus; VL, vastus lateralis; VM, vastus medialis; RF, rectus femoris; Glut-M, gluteus medius; CI, confidence interval.

The statistical analyses indicated significant main effects of “group” for VL activity (p<0.037; d = 0.57) during the swing phase of walking (Table 8). Pair-wise comparisons showed significantly larger VL activity (p = 0.037; d = 0.59) during the swing phase in PF compared with healthy controls (Table 8).

Finally, significant group by surface interactions were identified for RF activity (p<0.045; d = 0.55) during the swing phase of walking (Table 8). In the over PF group but not the healthy controls, significantly lower RF activities (p = 0.045, d = 0.51) were found when walking on sand compared with stable ground.

## Discussion

This study examined GRFs and activities of selected lower limb muscles in individuals with PF compared with healthy controls when walking at self-selected speed on sand versus stable ground.

The main findings of this study can be summarized as follows: i) Irrespective of the experimental group under consideration, longer stance times and times to peak were found for peak lateral GRFs during heel contact when walking on sand compared with walking on stable ground; ii) Irrespective of the group, slower gait speed, lower peak posterior GRFs were observed during heel contact, and lower peak anterior GRFs were found during the push off phase when walking on sand compared with walking on stable ground; iii) In the over PF group, lower peak positive FM amplitudes were found during the push off phase and lower vertical loading rates were observed during the loading phase when walking on sand compared with walking on stable ground; iv) PF showed lower loading rates during the loading phase compared with healthy controls; v) Irrespective of the group, comparable lower limb muscle activities were found during walking on sand compared with walking on stable ground during the loading and push off phases; vi) Irrespective of the group, higher Gas-M activities were observed during the mid-stance phase of walking on sand compared with walking on stable ground.

This study demonstrated significantly lower peak posterior GRF amplitudes during the loading phase, lower peak anterior GRF amplitudes during the push off phase, and shorter TTP for peak lateral GRF amplitudes in PF and healthy controls when walking on sand compared with stable ground. The observed reduction in peak anterior GRF amplitudes during the push off phase while walking on sand may be representative of foot and ankle instability due to impairment of the midtarsal locking mechanism in the late stance phase which provides an adequate lever to push off against. It has previously been reported that walking on stable ground compared with walking on sand results in a greater maximal braking force and maximal propulsive force in healthy male students aged 18–30 years [29]. Of note, we did not find any significant main effects of “surface” for peak medio-lateral GRFs. However, a previous study has demonstrated that walking on sand resulted in larger medio-lateral GRF in healthy male students aged 18–30 years [29].

In this study, lower peak positive FM amplitudes were found during the push off phase and lower vertical loading rates were observed during the loading phase when walking on sand compared with walking on stable ground in the over PF group. Notably, increased loading rates and impact shocks may constitute biomechanical risk factors for orthopedic injuries such as knee osteoarthritis or stress fractures [37, 38]. Therefore, a modified walking surface using sand could possibly reduce the injury risk for individuals with PF due to lower vertical loading rates and FM amplitudes.

With regards to EMG activities, no statistically significant main effects of “surface” were found for activities of selected lower limb muscles during the loading and the push off phases. Previous studies have demonstrated that when running on a stable surface, knee flexion increases during the loading response phase which is primarily due to eccentric loading of the quadriceps muscles during weight acceptance [39–42] followed by a concentric knee extensor action from mid-stance to push off [39, 41, 43]. In disagreement with our results, a recent study demonstrated greater net knee extensor activities (RF, VL, VM) when running on sand compared with stable ground [44]. On sand, the maximal hip abductor/adductor moments were greater than the value on stable ground [45]. However, our results did not demonstrate any significant changes in hip abductor muscle activity (Glut-M) when walking on sand compared with stable ground. This might be due to a slower gait speed while walking on sand compared with walking on stable ground. In agreement with our findings, a previous study reported longer stance times when running on sand compared with stable ground [44]. Of note, BF activity was significantly different when walking on sand compared with walking on stable ground. In contrast to our findings, a previous study demonstrated that during the stance phase of running at similar speed on sand and stable ground, hamstrings activation (i.e., semimembranosus and BF) was twice as high on sand [44]. With reference to the literature [44], running on sand produced muscle activation levels that were 65% and 30% higher in the quadriceps muscle (RF, VL, VM) during the stance phase of running at 8 and 11 km.h-1 compared to running on stable ground. A previous study suggested that the increased energy cost of running on sand can partly be attributed to the increased EMG activity which is associated with greater hip and knee ranges of motion compared with stable surface running [44].

In this study, we found significant main effects of “surface” for Gas-M activity during the mid-stance phase. Pair-wise comparisons revealed significantly greater Gas-M activity when walking on sand compared with walking on stable ground. A recent study showed that the thickness of the RF was significantly smaller in PF individuals compared to healthy peers [16]. In contrast, Ashnagar reported that VL and VM muscle thickness were not different in young adults with PF compared with healthy controls [16]. To compensate for these muscular deficits, higher Gas-M activities are needed to control the body and to prevent it from falling during push-off [46–47]. Of note, Gas-M plays a crucial role to generate propulsion during walking. The modified alignment of the rearfoot bones in association with the less stable foot articulations, and the internal rotation of the tibia during the early stage of the stance phase could be a possible reason for higher Gas-M activities [47]. The altered rearfoot bones configuration may result in modified muscle pulling forces. Therefore, in order to generate similar forces, higher muscle activities are required. Previous studies have shown that knee valgus during the early stance phase of PF individuals is associated with hip adduction and affords higher hip abductor activities, mainly of the Glut-M muscle [17]. Of note, some studies even showed weakness of the Glut-M muscles which act as hip abductor. This may increase the risk of sustaining injuries that are attributed to excessive subtalar pronation [12, 48–50]. During the heel strike of running or walking, the Glut-M contracts to maintain lower limbs alignment from the pelvis to the femur, knee, and tibia and finally the foot [7, 48]. Weakness of the Glut-M muscles causes hip adduction and consequently inward rotation of the femur, knee, and tibia [7, 49, 50]. This excessive inward rotation of the leg is related to an increased foot pronation [7, 12, 49, 50]. The foot muscles that control pronation are not strong enough to counteract these forces from the hip and lower leg. As a consequence, over pronation occurs which may lead to subsequent injuries due to overload and malalignment [51]. There is evidence that running on sand may reduce over PF during the stance phase and might therefore lower the risk of sustaining injuries during running [44].

In this study, we showed similar VL activity during the mid-stance and push off phases together with lower VL activity during the swing phase when walking on sand compared with walking on stable ground. There is evidence that running on sand at relatively slow velocities produced similar hip and knee flexion angles compared with running at faster velocities on stable ground [44]. Pinnington and colleagues [44] reported similar kinematics but differences in activities of muscles that control the hip and knee joint during the stance and swing phases of the running cycle when running on sand compared with stable ground.

This study has a few limitations that should be discussed. First, the number of study participants was relatively small. However, we conducted an a priori power analysis and the findings support our initial cohort size. Second, we did not record kinematic data in this study. This should be done in future research. Third, we examined the acute effects of walking on sand versus stable ground. Future studies are needed to examine the long-term effects of walking on sand to establish whether sand is suitable as a preventive/rehabilitative means to treat foot over pronation.

## Conclusions

The observed lower velocities during walking on sand compared with stable ground were accompanied by lower peak positive free moments during the push off phase and loading rates during the loading phase. Our findings of similar lower limb muscle activities during walking on sand compared with walking on stable ground in the PF group together with lower free moment amplitudes, vertical loading rates, and lower walking velocities on sand may indicate more relative muscle activity on sand compared with stable ground. There is evidence [52] of greater muscle activity with faster walking speed. Therefore, our finding of similar muscle activities while walking on sand compared with stable ground together with slower walking speed on sand is indicative of a higher relative muscle activity on sand. However, further research is needed to verify our findings.

## Supporting information

S1 File Log files of the statistical analyses. (DOCX)
