# Supplementary material for: Ground reaction forces and muscle activity while walking on sand versus stable ground in individuals with pronated feet compared with healthy controls
Source: PLoS One. 2019 Sep 26;14(9):e0223219. doi: 10.1371/journal.pone.0223219 (PMC6762175; doi:10.1371/journal.pone.0223219)
Supplement: S1 File — (DOCX) [file pone.0223219.s001.docx]

**Free moment**

| **Descriptive Statistics** | | | | |
| --- | --- | --- | --- | --- |
|  | group | Mean | Std. Deviation | N |
| levelFM1 | pronated | 2.337777 | 1.4561433 | 30 |
|  | healthy | 2.360800 | .9954168 | 29 |
|  | Total | 2.349093 | 1.2404253 | 59 |
| SandFM1 | pronated | 2.226767 | 1.1211851 | 30 |
|  | healthy | 2.569686 | .9512964 | 29 |
|  | Total | 2.395320 | 1.0465679 | 59 |

| **Tests of Within-Subjects Effects** | | | | | | | | | |
| --- | --- | --- | --- | --- | --- | --- | --- | --- | --- |
| Measure: MEASURE_1 | | | | | | | | | |
| Source | | Type III Sum of Squares | df | Mean Square | F | Sig. | Partial Eta Squared | Noncent. Parameter | Observed Power^a^ |
| surface | Sphericity Assumed | 3.751 | 1 | 3.751 | 7.163 | .010 | .113 | 7.163 | .749 |
|  | Greenhouse-Geisser | 3.751 | 1.000 | 3.751 | 7.163 | .010 | .113 | 7.163 | .749 |
|  | Huynh-Feldt | 3.751 | 1.000 | 3.751 | 7.163 | .010 | .113 | 7.163 | .749 |
|  | Lower-bound | 3.751 | 1.000 | 3.751 | 7.163 | .010 | .113 | 7.163 | .749 |
| surface * group | Sphericity Assumed | 2.601 | 1 | 2.601 | 4.967 | .030 | .081 | 4.967 | .591 |
|  | Greenhouse-Geisser | 2.601 | 1.000 | 2.601 | 4.967 | .030 | .081 | 4.967 | .591 |
|  | Huynh-Feldt | 2.601 | 1.000 | 2.601 | 4.967 | .030 | .081 | 4.967 | .591 |
|  | Lower-bound | 2.601 | 1.000 | 2.601 | 4.967 | .030 | .081 | 4.967 | .591 |
| Error(surface) | Sphericity Assumed | 29.327 | 56 | .524 |  |  |  |  |  |
|  | Greenhouse-Geisser | 29.327 | 56.000 | .524 |  |  |  |  |  |
|  | Huynh-Feldt | 29.327 | 56.000 | .524 |  |  |  |  |  |
|  | Lower-bound | 29.327 | 56.000 | .524 |  |  |  |  |  |
| a. Computed using alpha = .05 | | | | | | | | | |

| **Tests of Between-Subjects Effects** | | | | | | | | |
| --- | --- | --- | --- | --- | --- | --- | --- | --- |
| Measure: MEASURE_1 | | | | | | | | |
| Transformed Variable: Average | | | | | | | | |
| Source | Type III Sum of Squares | df | Mean Square | F | Sig. | Partial Eta Squared | Noncent. Parameter | Observed Power^a^ |
| Intercept | 3.444 | 1 | 3.444 | 2.383 | .128 | .041 | 2.383 | .329 |
| group | 2.834 | 1 | 2.834 | 1.961 | .167 | .034 | 1.961 | .280 |
| Error | 80.939 | 56 | 1.445 |  |  |  |  |  |
| a. Computed using alpha = .05 | | | | | | | | |

| **Descriptive Statistics** | | | | |
| --- | --- | --- | --- | --- |
|  | group | Mean | Std. Deviation | N |
| levelFM2 | pronated | -.881603 | .6201193 | 30 |
|  | healthy | -.741334 | .9765253 | 29 |
|  | Total | -.812658 | .8109473 | 59 |
| SandFM2 | pronated | -.902577 | .6809600 | 30 |
|  | healthy | -1.069045 | .9888047 | 29 |
|  | Total | -.984400 | .8431539 | 59 |

| **Tests of Within-Subjects Effects** | | | | | | | | | |
| --- | --- | --- | --- | --- | --- | --- | --- | --- | --- |
| Measure: MEASURE_1 | | | | | | | | | |
| Source | | Type III Sum of Squares | df | Mean Square | F | Sig. | Partial Eta Squared | Noncent. Parameter | Observed Power^a^ |
| surface | Sphericity Assumed | 1.048 | 1 | 1.048 | 2.213 | .142 | .038 | 2.213 | .310 |
|  | Greenhouse-Geisser | 1.048 | 1.000 | 1.048 | 2.213 | .142 | .038 | 2.213 | .310 |
|  | Huynh-Feldt | 1.048 | 1.000 | 1.048 | 2.213 | .142 | .038 | 2.213 | .310 |
|  | Lower-bound | 1.048 | 1.000 | 1.048 | 2.213 | .142 | .038 | 2.213 | .310 |
|  | Lower-bound | .604 | 1.000 | .604 | 1.277 | .263 | .022 | 1.277 | .199 |
| surface * group | Sphericity Assumed | 1.176 | 1 | 1.176 | 2.483 | .121 | .042 | 2.483 | .341 |
|  | Greenhouse-Geisser | 1.176 | 1.000 | 1.176 | 2.483 | .121 | .042 | 2.483 | .341 |
|  | Huynh-Feldt | 1.176 | 1.000 | 1.176 | 2.483 | .121 | .042 | 2.483 | .341 |
|  | Lower-bound | 1.176 | 1.000 | 1.176 | 2.483 | .121 | .042 | 2.483 | .341 |
| Error(surface) | Sphericity Assumed | 26.508 | 56 | .473 |  |  |  |  |  |
|  | Greenhouse-Geisser | 26.508 | 56.000 | .473 |  |  |  |  |  |
|  | Huynh-Feldt | 26.508 | 56.000 | .473 |  |  |  |  |  |
|  | Lower-bound | 26.508 | 56.000 | .473 |  |  |  |  |  |
| a. Computed using alpha = .05 | | | | | | | | | |

| **Tests of Between-Subjects Effects** | | | | | | | | |
| --- | --- | --- | --- | --- | --- | --- | --- | --- |
| Measure: MEASURE_1 | | | | | | | | |
| Transformed Variable: Average | | | | | | | | |
| Source | Type III Sum of Squares | df | Mean Square | F | Sig. | Partial Eta Squared | Noncent. Parameter | Observed Power^a^ |
| Intercept | .085 | 1 | .085 | .108 | .743 | .002 | .108 | .062 |
| group | 1.201 | 1 | 1.201 | 1.525 | .222 | .027 | 1.525 | .229 |
| Error | 44.101 | 56 | .788 |  |  |  |  |  |
| a. Computed using alpha = .05 | | | | | | | | |

**Medio-lateral ground reaction force**

| **Descriptive Statistics** | | | | |
| --- | --- | --- | --- | --- |
|  | group | Mean | Std. Deviation | N |
| levelFx1 | pronated | 7.9843 | 3.11876 | 30 |
|  | healthy | 7.4514 | 2.92243 | 29 |
|  | Total | 7.7224 | 3.00975 | 59 |
| SandFx1 | pronated | 7.7927 | 3.16930 | 30 |
|  | healthy | 7.0355 | 3.85686 | 29 |
|  | Total | 7.4205 | 3.51414 | 59 |

| **Tests of Within-Subjects Effects** | | | | | | | | | | |
| --- | --- | --- | --- | --- | --- | --- | --- | --- | --- | --- |
| Measure: MEASURE_1 | | | | | | | | | | |
| Source | | Type III Sum of Squares | df | Mean Square | F | Sig. | Partial Eta Squared | Noncent. Parameter | | Observed Power^a^ |
| surface | Sphericity Assumed | 21.939 | 1 | 21.939 | 2.677 | .107 | .046 | 2.677 | .363 | |
|  | Greenhouse-Geisser | 21.939 | 1.000 | 21.939 | 2.677 | .107 | .046 | 2.677 | | .363 |
|  | Huynh-Feldt | 21.939 | 1.000 | 21.939 | 2.677 | .107 | .046 | 2.677 | | .363 |
|  | Lower-bound | 21.939 | 1.000 | 21.939 | 2.677 | .107 | .046 | 2.677 | | .363 |
| surface * group | Sphericity Assumed | 3.065 | 1 | 3.065 | .374 | .543 | .007 | .374 | | .092 |
|  | Greenhouse-Geisser | 3.065 | 1.000 | 3.065 | .374 | .543 | .007 | .374 | | .092 |
|  | Huynh-Feldt | 3.065 | 1.000 | 3.065 | .374 | .543 | .007 | .374 | | .092 |
|  | Lower-bound | 3.065 | 1.000 | 3.065 | .374 | .543 | .007 | .374 | | .092 |
| Error(surface) | Sphericity Assumed | 458.946 | 56 | 8.195 |  |  |  |  | |  |
|  | Greenhouse-Geisser | 458.946 | 56.000 | 8.195 |  |  |  |  | |  |
|  | Huynh-Feldt | 458.946 | 56.000 | 8.195 |  |  |  |  | |  |
|  | Lower-bound | 458.946 | 56.000 | 8.195 |  |  |  |  | |  |
| a. Computed using alpha = .05 | | | | | | | | | | |

| **Tests of Between-Subjects Effects** | | | | | | | | |
| --- | --- | --- | --- | --- | --- | --- | --- | --- |
| Measure: MEASURE_1 | | | | | | | | |
| Transformed Variable: Average | | | | | | | | |
| Source | Type III Sum of Squares | df | Mean Square | F | Sig. | Partial Eta Squared | Noncent. Parameter | Observed Power^a^ |
| Intercept | 764.309 | 1 | 764.309 | 58.689 | .000 | .512 | 58.689 | 1.000 |
| group | 3.016 | 1 | 3.016 | .232 | .632 | .004 | .232 | .076 |
| Error | 729.290 | 56 | 13.023 |  |  |  |  |  |
| a. Computed using alpha = .05 | | | | | | | | |

| **Descriptive Statistics** | | | | |
| --- | --- | --- | --- | --- |
|  | group | Mean | Std. Deviation | N |
| levelFx2 | pronated | -5.4093 | 3.13226 | 30 |
|  | healthy | -6.7576 | 3.88904 | 29 |
|  | Total | -6.0720 | 3.55938 | 59 |
| SandFx2 | pronated | -7.0700 | 4.79744 | 30 |
|  | healthy | -9.0517 | 5.07769 | 29 |
|  | Total | -8.0441 | 4.99531 | 59 |

| **Tests of Within-Subjects Effects** | | | | | | | | | |
| --- | --- | --- | --- | --- | --- | --- | --- | --- | --- |
| Measure: MEASURE_1 | | | | | | | | | |
| Source | | Type III Sum of Squares | df | Mean Square | F | Sig. | Partial Eta Squared | Noncent. Parameter | Observed Power^a^ |
| surface | Sphericity Assumed | .025 | 1 | .025 | .002 | .964 | .000 | .002 | .050 |
|  | Greenhouse-Geisser | .025 | 1.000 | .025 | .002 | .964 | .000 | .002 | .050 |
|  | Huynh-Feldt | .025 | 1.000 | .025 | .002 | .964 | .000 | .002 | .050 |
|  | Lower-bound | .025 | 1.000 | .025 | .002 | .964 | .000 | .002 | .050 |
| surface * group | Sphericity Assumed | .043 | 1 | .043 | .004 | .952 | .000 | .004 | .050 |
|  | Greenhouse-Geisser | .043 | 1.000 | .043 | .004 | .952 | .000 | .004 | .050 |
|  | Huynh-Feldt | .043 | 1.000 | .043 | .004 | .952 | .000 | .004 | .050 |
|  | Lower-bound | .043 | 1.000 | .043 | .004 | .952 | .000 | .004 | .050 |
| Error(surface) | Sphericity Assumed | 662.021 | 56 | 11.822 |  |  |  |  |  |
|  | Greenhouse-Geisser | 662.021 | 56.000 | 11.822 |  |  |  |  |  |
|  | Huynh-Feldt | 662.021 | 56.000 | 11.822 |  |  |  |  |  |
|  | Lower-bound | 662.021 | 56.000 | 11.822 |  |  |  |  |  |
| a. Computed using alpha = .05 | | | | | | | | | |

| **Tests of Between-Subjects Effects** | | | | | | | | |
| --- | --- | --- | --- | --- | --- | --- | --- | --- |
| Measure: MEASURE_1 | | | | | | | | |
| Transformed Variable: Average | | | | | | | | |
| Source | Type III Sum of Squares | df | Mean Square | F | Sig. | Partial Eta Squared | Noncent. Parameter | Observed Power^a^ |
| Intercept | 325.591 | 1 | 325.591 | 13.022 | .001 | .189 | 13.022 | .944 |
| group | 36.696 | 1 | 36.696 | 1.468 | .231 | .026 | 1.468 | .222 |
| Error | 1400.163 | 56 | 25.003 |  |  |  |  |  |
| a. Computed using alpha = .05 | | | | | | | | |

**Antro-posterior ground reaction force**

| **Descriptive Statistics** | | | | |
| --- | --- | --- | --- | --- |
|  | group | Mean | Std. Deviation | N |
| levelFy1 | pronated | -7.8077 | 6.73077 | 30 |
|  | healthy | -4.6538 | 5.40909 | 29 |
|  | Total | -6.2575 | 6.26938 | 59 |
| SandFy1 | pronated | -3.4543 | 3.00755 | 30 |
|  | healthy | -3.6283 | 1.33894 | 29 |
|  | Total | -3.5398 | 2.32290 | 59 |

| **Tests of Within-Subjects Effects** | | | | | | | | | |
| --- | --- | --- | --- | --- | --- | --- | --- | --- | --- |
| Measure: MEASURE_1 | | | | | | | | | |
| Source | | Type III Sum of Squares | df | Mean Square | F | Sig. | Partial Eta Squared | Noncent. Parameter | Observed Power^a^ |
| surface | Sphericity Assumed | 144.105 | 1 | 144.105 | 8.464 | .005 | .131 | 8.464 | .816 |
|  | Greenhouse-Geisser | 144.105 | 1.000 | 144.105 | 8.464 | .005 | .131 | 8.464 | .816 |
|  | Huynh-Feldt | 144.105 | 1.000 | 144.105 | 8.464 | .005 | .131 | 8.464 | .816 |
|  | Lower-bound | 144.105 | 1.000 | 144.105 | 8.464 | .005 | .131 | 8.464 | .816 |
| surface * group | Sphericity Assumed | 22.760 | 1 | 22.760 | 1.337 | .252 | .023 | 1.337 | .206 |
|  | Greenhouse-Geisser | 22.760 | 1.000 | 22.760 | 1.337 | .252 | .023 | 1.337 | .206 |
|  | Huynh-Feldt | 22.760 | 1.000 | 22.760 | 1.337 | .252 | .023 | 1.337 | .206 |
|  | Lower-bound | 22.760 | 1.000 | 22.760 | 1.337 | .252 | .023 | 1.337 | .206 |
| Error(surface) | Sphericity Assumed | 953.418 | 56 | 17.025 |  |  |  |  |  |
|  | Greenhouse-Geisser | 953.418 | 56.000 | 17.025 |  |  |  |  |  |
|  | Huynh-Feldt | 953.418 | 56.000 | 17.025 |  |  |  |  |  |
|  | Lower-bound | 953.418 | 56.000 | 17.025 |  |  |  |  |  |
| a. Computed using alpha = .05 | | | | | | | | | |

| **Tests of Between-Subjects Effects** | | | | | | | | |
| --- | --- | --- | --- | --- | --- | --- | --- | --- |
| Measure: MEASURE_1 | | | | | | | | |
| Transformed Variable: Average | | | | | | | | |
| Source | Type III Sum of Squares | df | Mean Square | F | Sig. | Partial Eta Squared | Noncent. Parameter | Observed Power^a^ |
| Intercept | 579.437 | 1 | 579.437 | 23.998 | .000 | .300 | 23.998 | .998 |
| group | 13.194 | 1 | 13.194 | .546 | .463 | .010 | .546 | .112 |
| Error | 1352.119 | 56 | 24.145 |  |  |  |  |  |
| a. Computed using alpha = .05 | | | | | | | | |

| **Descriptive Statistics** | | | | |
| --- | --- | --- | --- | --- |
|  | group | Mean | Std. Deviation | N |
| levelFy2 | pronated | 8.4633 | 5.51711 | 30 |
|  | healthy | 5.8493 | 5.32392 | 29 |
|  | Total | 7.1785 | 5.53533 | 59 |
| SandFy2 | pronated | 4.6283 | 2.31020 | 30 |
|  | healthy | 4.0631 | 1.44773 | 29 |
|  | Total | 4.3505 | 1.93947 | 59 |

| **Tests of Within-Subjects Effects** | | | | | | | | | |
| --- | --- | --- | --- | --- | --- | --- | --- | --- | --- |
| Measure: MEASURE_1 | | | | | | | | | |
| Source | | Type III Sum of Squares | df | Mean Square | F | Sig. | Partial Eta Squared | Noncent. Parameter | Observed Power^a^ |
| surface | Sphericity Assumed | 188.787 | 1 | 188.787 | 12.479 | .001 | .182 | 12.479 | .935 |
|  | Greenhouse-Geisser | 188.787 | 1.000 | 188.787 | 12.479 | .001 | .182 | 12.479 | .935 |
|  | Huynh-Feldt | 188.787 | 1.000 | 188.787 | 12.479 | .001 | .182 | 12.479 | .935 |
|  | Lower-bound | 188.787 | 1.000 | 188.787 | 12.479 | .001 | .182 | 12.479 | .935 |
| surface * group | Sphericity Assumed | .887 | 1 | .887 | .059 | .810 | .001 | .059 | .057 |
|  | Greenhouse-Geisser | .887 | 1.000 | .887 | .059 | .810 | .001 | .059 | .057 |
|  | Huynh-Feldt | .887 | 1.000 | .887 | .059 | .810 | .001 | .059 | .057 |
|  | Lower-bound | .887 | 1.000 | .887 | .059 | .810 | .001 | .059 | .057 |
| Error(surface) | Sphericity Assumed | 847.218 | 56 | 15.129 |  |  |  |  |  |
|  | Greenhouse-Geisser | 847.218 | 56.000 | 15.129 |  |  |  |  |  |
|  | Huynh-Feldt | 847.218 | 56.000 | 15.129 |  |  |  |  |  |
|  | Lower-bound | 847.218 | 56.000 | 15.129 |  |  |  |  |  |
| a. Computed using alpha = .05 | | | | | | | | | |

| **Tests of Between-Subjects Effects** | | | | | | | | |
| --- | --- | --- | --- | --- | --- | --- | --- | --- |
| Measure: MEASURE_1 | | | | | | | | |
| Transformed Variable: Average | | | | | | | | |
| Source | Type III Sum of Squares | df | Mean Square | F | Sig. | Partial Eta Squared | Noncent. Parameter | Observed Power^a^ |
| Intercept | 882.211 | 1 | 882.211 | 60.724 | .000 | .520 | 60.724 | 1.000 |
| group | 8.177 | 1 | 8.177 | .563 | .456 | .010 | .563 | .114 |
| Error | 813.574 | 56 | 14.528 |  |  |  |  |  |
| a. Computed using alpha = .05 | | | | | | | | |

**Vertical ground reaction force**

| **Descriptive Statistics** | | | | |
| --- | --- | --- | --- | --- |
|  | group | Mean | Std. Deviation | N |
| levelFz1 | pronated | 110.5127 | 18.85484 | 30 |
|  | healthy | 103.5303 | 7.06578 | 29 |
|  | Total | 107.0807 | 14.63725 | 59 |
| SandFz1 | pronated | 107.1180 | 16.15561 | 30 |
|  | healthy | 102.0824 | 11.56794 | 29 |
|  | Total | 104.6429 | 14.19683 | 59 |

| **Tests of Within-Subjects Effects** | | | | | | | | | |
| --- | --- | --- | --- | --- | --- | --- | --- | --- | --- |
| Measure: MEASURE_1 | | | | | | | | | |
| Source | | Type III Sum of Squares | df | Mean Square | F | Sig. | Partial Eta Squared | Noncent. Parameter | Observed Power^a^ |
| surface | Sphericity Assumed | 123.196 | 1 | 123.196 | 1.743 | .192 | .030 | 1.743 | .254 |
|  | Greenhouse-Geisser | 123.196 | 1.000 | 123.196 | 1.743 | .192 | .030 | 1.743 | .254 |
|  | Huynh-Feldt | 123.196 | 1.000 | 123.196 | 1.743 | .192 | .030 | 1.743 | .254 |
|  | Lower-bound | 123.196 | 1.000 | 123.196 | 1.743 | .192 | .030 | 1.743 | .254 |
| surface * group | Sphericity Assumed | 2.515 | 1 | 2.515 | .036 | .851 | .001 | .036 | .054 |
|  | Greenhouse-Geisser | 2.515 | 1.000 | 2.515 | .036 | .851 | .001 | .036 | .054 |
|  | Huynh-Feldt | 2.515 | 1.000 | 2.515 | .036 | .851 | .001 | .036 | .054 |
|  | Lower-bound | 2.515 | 1.000 | 2.515 | .036 | .851 | .001 | .036 | .054 |
| Error(surface) | Sphericity Assumed | 3958.824 | 56 | 70.693 |  |  |  |  |  |
|  | Greenhouse-Geisser | 3958.824 | 56.000 | 70.693 |  |  |  |  |  |
|  | Huynh-Feldt | 3958.824 | 56.000 | 70.693 |  |  |  |  |  |
|  | Lower-bound | 3958.824 | 56.000 | 70.693 |  |  |  |  |  |
| a. Computed using alpha = .05 | | | | | | | | | |

| **Tests of Between-Subjects Effects** | | | | | | | | |
| --- | --- | --- | --- | --- | --- | --- | --- | --- |
| Measure: MEASURE_1 | | | | | | | | |
| Transformed Variable: Average | | | | | | | | |
| Source | Type III Sum of Squares | df | Mean Square | F | Sig. | Partial Eta Squared | Noncent. Parameter | Observed Power^a^ |
| Intercept | 143960.834 | 1 | 143960.834 | 462.436 | .000 | .892 | 462.436 | 1.000 |
| group | 160.935 | 1 | 160.935 | .517 | .475 | .009 | .517 | .109 |
| Error | 17433.360 | 56 | 311.310 |  |  |  |  |  |
| a. Computed using alpha = .05 | | | | | | | | |

| **Descriptive Statistics** | | | | |
| --- | --- | --- | --- | --- |
|  | group | Mean | Std. Deviation | N |
| levelFz2 | pronated | 78.5683 | 11.36902 | 30 |
|  | healthy | 79.5279 | 7.43800 | 29 |
|  | Total | 79.0400 | 9.56919 | 59 |
| SandFz2 | pronated | 80.1343 | 6.50331 | 30 |
|  | healthy | 77.3393 | 9.87591 | 29 |
|  | Total | 78.7605 | 8.37961 | 59 |

| **Tests of Within-Subjects Effects** | | | | | | | | | |
| --- | --- | --- | --- | --- | --- | --- | --- | --- | --- |
| Measure: MEASURE_1 | | | | | | | | | |
| Source | | Type III Sum of Squares | df | Mean Square | F | Sig. | Partial Eta Squared | Noncent. Parameter | Observed Power^a^ |
| surface | Sphericity Assumed | 26.534 | 1 | 26.534 | .640 | .427 | .011 | .640 | .123 |
|  | Greenhouse-Geisser | 26.534 | 1.000 | 26.534 | .640 | .427 | .011 | .640 | .123 |
|  | Huynh-Feldt | 26.534 | 1.000 | 26.534 | .640 | .427 | .011 | .640 | .123 |
|  | Lower-bound | 26.534 | 1.000 | 26.534 | .640 | .427 | .011 | .640 | .123 |
| surface * group | Sphericity Assumed | 45.082 | 1 | 45.082 | 1.088 | .301 | .019 | 1.088 | .176 |
|  | Greenhouse-Geisser | 45.082 | 1.000 | 45.082 | 1.088 | .301 | .019 | 1.088 | .176 |
|  | Huynh-Feldt | 45.082 | 1.000 | 45.082 | 1.088 | .301 | .019 | 1.088 | .176 |
|  | Lower-bound | 45.082 | 1.000 | 45.082 | 1.088 | .301 | .019 | 1.088 | .176 |
| Error(surface) | Sphericity Assumed | 2320.647 | 56 | 41.440 |  |  |  |  |  |
|  | Greenhouse-Geisser | 2320.647 | 56.000 | 41.440 |  |  |  |  |  |
|  | Huynh-Feldt | 2320.647 | 56.000 | 41.440 |  |  |  |  |  |
|  | Lower-bound | 2320.647 | 56.000 | 41.440 |  |  |  |  |  |
| a. Computed using alpha = .05 | | | | | | | | | |

| **Tests of Between-Subjects Effects** | | | | | | | | |
| --- | --- | --- | --- | --- | --- | --- | --- | --- |
| Measure: MEASURE_1 | | | | | | | | |
| Transformed Variable: Average | | | | | | | | |
| Source | Type III Sum of Squares | df | Mean Square | F | Sig. | Partial Eta Squared | Noncent. Parameter | Observed Power^a^ |
| Intercept | 55219.995 | 1 | 55219.995 | 477.553 | .000 | .895 | 477.553 | 1.000 |
| group | 175.744 | 1 | 175.744 | 1.520 | .223 | .026 | 1.520 | .228 |
| Error | 6475.339 | 56 | 115.631 |  |  |  |  |  |
| a. Computed using alpha = .05 | | | | | | | | |

| **Descriptive Statistics** | | | | |
| --- | --- | --- | --- | --- |
|  | group | Mean | Std. Deviation | N |
| levelFz3 | pronated | 109.3687 | 16.27728 | 30 |
|  | healthy | 107.3969 | 5.86123 | 29 |
|  | Total | 108.3995 | 12.24941 | 59 |
| SandFz3 | pronated | 107.7853 | 14.60646 | 30 |
|  | healthy | 105.3597 | 7.51059 | 29 |
|  | Total | 106.5931 | 11.63624 | 59 |

| **Tests of Within-Subjects Effects** | | | | | | | | | |
| --- | --- | --- | --- | --- | --- | --- | --- | --- | --- |
| Measure: MEASURE_1 | | | | | | | | | |
| Source | | Type III Sum of Squares | df | Mean Square | F | Sig. | Partial Eta Squared | Noncent. Parameter | Observed Power^a^ |
| surface | Sphericity Assumed | 51.626 | 1 | 51.626 | 2.748 | .103 | .047 | 2.748 | .371 |
|  | Greenhouse-Geisser | 51.626 | 1.000 | 51.626 | 2.748 | .103 | .047 | 2.748 | .371 |
|  | Huynh-Feldt | 51.626 | 1.000 | 51.626 | 2.748 | .103 | .047 | 2.748 | .371 |
|  | Lower-bound | 51.626 | 1.000 | 51.626 | 2.748 | .103 | .047 | 2.748 | .371 |
| surface * group | Sphericity Assumed | 9.075 | 1 | 9.075 | .483 | .490 | .009 | .483 | .105 |
|  | Greenhouse-Geisser | 9.075 | 1.000 | 9.075 | .483 | .490 | .009 | .483 | .105 |
|  | Huynh-Feldt | 9.075 | 1.000 | 9.075 | .483 | .490 | .009 | .483 | .105 |
|  | Lower-bound | 9.075 | 1.000 | 9.075 | .483 | .490 | .009 | .483 | .105 |
| Error(surface) | Sphericity Assumed | 1052.174 | 56 | 18.789 |  |  |  |  |  |
|  | Greenhouse-Geisser | 1052.174 | 56.000 | 18.789 |  |  |  |  |  |
|  | Huynh-Feldt | 1052.174 | 56.000 | 18.789 |  |  |  |  |  |
|  | Lower-bound | 1052.174 | 56.000 | 18.789 |  |  |  |  |  |
| a. Computed using alpha = .05 | | | | | | | | | |

| **Tests of Between-Subjects Effects** | | | | | | | | |
| --- | --- | --- | --- | --- | --- | --- | --- | --- |
| Measure: MEASURE_1 | | | | | | | | |
| Transformed Variable: Average | | | | | | | | |
| Source | Type III Sum of Squares | df | Mean Square | F | Sig. | Partial Eta Squared | Noncent. Parameter | Observed Power^a^ |
| Intercept | 132719.748 | 1 | 132719.748 | 495.222 | .000 | .898 | 495.222 | 1.000 |
| group | 9.433 | 1 | 9.433 | .035 | .852 | .001 | .035 | .054 |
| Error | 15008.034 | 56 | 268.001 |  |  |  |  |  |
| a. Computed using alpha = .05 | | | | | | | | |

| **Descriptive Statistics** | | | | |
| --- | --- | --- | --- | --- |
|  | group | Mean | Std. Deviation | N |
| levelimpX | pronated | 3.1827 | 2.22506 | 30 |
|  | healthy | 3.5259 | 1.85885 | 29 |
|  | Total | 3.3514 | 2.04291 | 59 |
| SandimpX | pronated | 4.1210 | 3.29733 | 30 |
|  | healthy | 5.7790 | 3.17497 | 29 |
|  | Total | 4.9359 | 3.31685 | 59 |

| **Tests of Within-Subjects Effects** | | | | | | | | | |
| --- | --- | --- | --- | --- | --- | --- | --- | --- | --- |
| Measure: MEASURE_1 | | | | | | | | | |
| Source | | Type III Sum of Squares | df | Mean Square | F | Sig. | Partial Eta Squared | Noncent. Parameter | Observed Power^a^ |
| surface | Sphericity Assumed | .519 | 1 | .519 | .080 | .778 | .001 | .080 | .059 |
|  | Greenhouse-Geisser | .519 | 1.000 | .519 | .080 | .778 | .001 | .080 | .059 |
|  | Huynh-Feldt | .519 | 1.000 | .519 | .080 | .778 | .001 | .080 | .059 |
|  | Lower-bound | .519 | 1.000 | .519 | .080 | .778 | .001 | .080 | .059 |
| surface * group | Sphericity Assumed | 3.118 | 1 | 3.118 | .483 | .490 | .009 | .483 | .105 |
|  | Greenhouse-Geisser | 3.118 | 1.000 | 3.118 | .483 | .490 | .009 | .483 | .105 |
|  | Huynh-Feldt | 3.118 | 1.000 | 3.118 | .483 | .490 | .009 | .483 | .105 |
|  | Lower-bound | 3.118 | 1.000 | 3.118 | .483 | .490 | .009 | .483 | .105 |
| Error(surface) | Sphericity Assumed | 361.233 | 56 | 6.451 |  |  |  |  |  |
|  | Greenhouse-Geisser | 361.233 | 56.000 | 6.451 |  |  |  |  |  |
|  | Huynh-Feldt | 361.233 | 56.000 | 6.451 |  |  |  |  |  |
|  | Lower-bound | 361.233 | 56.000 | 6.451 |  |  |  |  |  |
| a. Computed using alpha = .05 | | | | | | | | | |

| **Tests of Between-Subjects Effects** | | | | | | | | |
| --- | --- | --- | --- | --- | --- | --- | --- | --- |
| Measure: MEASURE_1 | | | | | | | | |
| Transformed Variable: Average | | | | | | | | |
| Source | Type III Sum of Squares | df | Mean Square | F | Sig. | Partial Eta Squared | Noncent. Parameter | Observed Power^a^ |
| Intercept | 94.308 | 1 | 94.308 | 11.742 | .001 | .173 | 11.742 | .920 |
| group | 10.740 | 1 | 10.740 | 1.337 | .252 | .023 | 1.337 | .206 |
| Error | 449.786 | 56 | 8.032 |  |  |  |  |  |
| a. Computed using alpha = .05 | | | | | | | | |

**Antro-posterior Impulse**

| **Descriptive Statistics** | | | | |
| --- | --- | --- | --- | --- |
|  | group | Mean | Std. Deviation | N |
| levelimpY | pronated | 3.4567 | 3.81642 | 30 |
|  | healthy | 2.2534 | 1.26320 | 29 |
|  | Total | 2.8653 | 2.90188 | 59 |
| SandimpY | pronated | 2.3397 | 3.24706 | 30 |
|  | healthy | 1.9652 | 1.62013 | 29 |
|  | Total | 2.1556 | 2.56408 | 59 |

| **Tests of Within-Subjects Effects** | | | | | | | | | |
| --- | --- | --- | --- | --- | --- | --- | --- | --- | --- |
| Measure: MEASURE_1 | | | | | | | | | |
| Source | | Type III Sum of Squares | df | Mean Square | F | Sig. | Partial Eta Squared | Noncent. Parameter | Observed Power^a^ |
| surface | Sphericity Assumed | 27.685 | 1 | 27.685 | 4.101 | .048 | .068 | 4.101 | .512 |
|  | Greenhouse-Geisser | 27.685 | 1.000 | 27.685 | 4.101 | .048 | .068 | 4.101 | .512 |
|  | Huynh-Feldt | 27.685 | 1.000 | 27.685 | 4.101 | .048 | .068 | 4.101 | .512 |
|  | Lower-bound | 27.685 | 1.000 | 27.685 | 4.101 | .048 | .068 | 4.101 | .512 |
| surface * group | Sphericity Assumed | .040 | 1 | .040 | .006 | .939 | .000 | .006 | .051 |
|  | Greenhouse-Geisser | .040 | 1.000 | .040 | .006 | .939 | .000 | .006 | .051 |
|  | Huynh-Feldt | .040 | 1.000 | .040 | .006 | .939 | .000 | .006 | .051 |
|  | Lower-bound | .040 | 1.000 | .040 | .006 | .939 | .000 | .006 | .051 |
| Error(surface) | Sphericity Assumed | 378.077 | 56 | 6.751 |  |  |  |  |  |
|  | Greenhouse-Geisser | 378.077 | 56.000 | 6.751 |  |  |  |  |  |
|  | Huynh-Feldt | 378.077 | 56.000 | 6.751 |  |  |  |  |  |
|  | Lower-bound | 378.077 | 56.000 | 6.751 |  |  |  |  |  |
| a. Computed using alpha = .05 | | | | | | | | | |

| **Tests of Between-Subjects Effects** | | | | | | | | |
| --- | --- | --- | --- | --- | --- | --- | --- | --- |
| Measure: MEASURE_1 | | | | | | | | |
| Transformed Variable: Average | | | | | | | | |
| Source | Type III Sum of Squares | df | Mean Square | F | Sig. | Partial Eta Squared | Noncent. Parameter | Observed Power^a^ |
| Intercept | 158.334 | 1 | 158.334 | 20.749 | .000 | .270 | 20.749 | .994 |
| group | 3.518 | 1 | 3.518 | .461 | .500 | .008 | .461 | .102 |
| Error | 427.326 | 56 | 7.631 |  |  |  |  |  |
| a. Computed using alpha = .05 | | | | | | | | |

**Vertical Impulse**

| **Descriptive Statistics** | | | | |
| --- | --- | --- | --- | --- |
|  | group | Mean | Std. Deviation | N |
| levelimpZ | pronated | 58.0830 | 6.67608 | 30 |
|  | healthy | 57.8690 | 8.71421 | 29 |
|  | Total | 57.9778 | 7.67829 | 59 |
| SandimpZ | pronated | 79.4427 | 105.08349 | 30 |
|  | healthy | 61.2121 | 16.66526 | 29 |
|  | Total | 70.4819 | 75.76176 | 59 |

| **Tests of Within-Subjects Effects** | | | | | | | | | |
| --- | --- | --- | --- | --- | --- | --- | --- | --- | --- |
| Measure: MEASURE_1 | | | | | | | | | |
| Source | | Type III Sum of Squares | df | Mean Square | F | Sig. | Partial Eta Squared | Noncent. Parameter | Observed Power^a^ |
| surface | Sphericity Assumed | 1775.598 | 1 | 1775.598 | .629 | .431 | .011 | .629 | .122 |
|  | Greenhouse-Geisser | 1775.598 | 1.000 | 1775.598 | .629 | .431 | .011 | .629 | .122 |
|  | Huynh-Feldt | 1775.598 | 1.000 | 1775.598 | .629 | .431 | .011 | .629 | .122 |
|  | Lower-bound | 1775.598 | 1.000 | 1775.598 | .629 | .431 | .011 | .629 | .122 |
| surface * group | Sphericity Assumed | 1184.008 | 1 | 1184.008 | .419 | .520 | .007 | .419 | .098 |
|  | Greenhouse-Geisser | 1184.008 | 1.000 | 1184.008 | .419 | .520 | .007 | .419 | .098 |
|  | Huynh-Feldt | 1184.008 | 1.000 | 1184.008 | .419 | .520 | .007 | .419 | .098 |
|  | Lower-bound | 1184.008 | 1.000 | 1184.008 | .419 | .520 | .007 | .419 | .098 |
| Error(surface) | Sphericity Assumed | 158149.068 | 56 | 2824.091 |  |  |  |  |  |
|  | Greenhouse-Geisser | 158149.068 | 56.000 | 2824.091 |  |  |  |  |  |
|  | Huynh-Feldt | 158149.068 | 56.000 | 2824.091 |  |  |  |  |  |
|  | Lower-bound | 158149.068 | 56.000 | 2824.091 |  |  |  |  |  |
| a. Computed using alpha = .05 | | | | | | | | | |

| **Tests of Between-Subjects Effects** | | | | | | | | |
| --- | --- | --- | --- | --- | --- | --- | --- | --- |
| Measure: MEASURE_1 | | | | | | | | |
| Transformed Variable: Average | | | | | | | | |
| Source | Type III Sum of Squares | df | Mean Square | F | Sig. | Partial Eta Squared | Noncent. Parameter | Observed Power^a^ |
| Intercept | 49037.787 | 1 | 49037.787 | 15.918 | .000 | .221 | 15.918 | .975 |
| group | 1516.584 | 1 | 1516.584 | .492 | .486 | .009 | .492 | .106 |
| Error | 172512.296 | 56 | 3080.577 |  |  |  |  |  |
| a. Computed using alpha = .05 | | | | | | | | |

| **Descriptive Statistics** | | | | |
| --- | --- | --- | --- | --- |
|  | group | Mean | Std. Deviation | N |
| LoadingrateL | pronated | 8.0605 | 4.82941 | 30 |
|  | healthy | 9.3357 | 17.33050 | 29 |
|  | Total | 8.6873 | 12.53275 | 59 |
| LoadingrateS | pronated | 7.6408 | 4.92741 | 30 |
|  | healthy | 9.9368 | 16.65640 | 29 |
|  | Total | 8.7693 | 12.14143 | 59 |

| **Tests of Within-Subjects Effects** | | | | | | | | | |
| --- | --- | --- | --- | --- | --- | --- | --- | --- | --- |
| Measure: MEASURE_1 | | | | | | | | | |
| Source | | Type III Sum of Squares | df | Mean Square | F | Sig. | Partial Eta Squared | Noncent. Parameter | Observed Power^a^ |
| surface | Sphericity Assumed | 1.227 | 1 | 1.227 | .008 | .929 | .000 | .008 | .051 |
|  | Greenhouse-Geisser | 1.227 | 1.000 | 1.227 | .008 | .929 | .000 | .008 | .051 |
|  | Huynh-Feldt | 1.227 | 1.000 | 1.227 | .008 | .929 | .000 | .008 | .051 |
|  | Lower-bound | 1.227 | 1.000 | 1.227 | .008 | .929 | .000 | .008 | .051 |
| surface * group | Sphericity Assumed | 8.626 | 1 | 8.626 | .056 | .815 | .001 | .056 | .056 |
|  | Greenhouse-Geisser | 8.626 | 1.000 | 8.626 | .056 | .815 | .001 | .056 | .056 |
|  | Huynh-Feldt | 8.626 | 1.000 | 8.626 | .056 | .815 | .001 | .056 | .056 |
|  | Lower-bound | 8.626 | 1.000 | 8.626 | .056 | .815 | .001 | .056 | .056 |
| Error(surface) | Sphericity Assumed | 8698.713 | 56 | 155.334 |  |  |  |  |  |
|  | Greenhouse-Geisser | 8698.713 | 56.000 | 155.334 |  |  |  |  |  |
|  | Huynh-Feldt | 8698.713 | 56.000 | 155.334 |  |  |  |  |  |
|  | Lower-bound | 8698.713 | 56.000 | 155.334 |  |  |  |  |  |
| a. Computed using alpha = .05 | | | | | | | | | |

| **Tests of Between-Subjects Effects** | | | | | | | | |
| --- | --- | --- | --- | --- | --- | --- | --- | --- |
| Measure: MEASURE_1 | | | | | | | | |
| Transformed Variable: Average | | | | | | | | |
| Source | Type III Sum of Squares | df | Mean Square | F | Sig. | Partial Eta Squared | Noncent. Parameter | Observed Power^a^ |
| Intercept | 4314.404 | 1 | 4314.404 | 33.010 | .000 | .371 | 33.010 | 1.000 |
| group | 647.432 | 1 | 647.432 | 4.954 | .030 | .081 | 4.954 | .590 |
| Error | 7319.257 | 56 | 130.701 |  |  |  |  |  |
| a. Computed using alpha = .05 | | | | | | | | |

**Time to peak for ground reaction forces**

| **Descriptive Statistics** | | | | |
| --- | --- | --- | --- | --- |
|  | group | Mean | Std. Deviation | N |
| levelTFx1 | pronated | 22.2667 | 11.47090 | 30 |
|  | healthy | 19.1414 | 16.75482 | 29 |
|  | Total | 20.7305 | 14.27571 | 59 |
| SandTFx1 | pronated | 25.5890 | 19.06250 | 30 |
|  | healthy | 12.3637 | 11.43757 | 29 |
|  | Total | 19.0884 | 17.00916 | 59 |

| **Tests of Within-Subjects Effects** | | | | | | | | | |
| --- | --- | --- | --- | --- | --- | --- | --- | --- | --- |
| Measure: MEASURE_1 | | | | | | | | | |
| Source | | Type III Sum of Squares | df | Mean Square | F | Sig. | Partial Eta Squared | Noncent. Parameter | Observed Power^a^ |
| surface | Sphericity Assumed | 789.269 | 1 | 789.269 | 4.235 | .044 | .070 | 4.235 | .525 |
|  | Greenhouse-Geisser | 789.269 | 1.000 | 789.269 | 4.235 | .044 | .070 | 4.235 | .525 |
|  | Huynh-Feldt | 789.269 | 1.000 | 789.269 | 4.235 | .044 | .070 | 4.235 | .525 |
|  | Lower-bound | 789.269 | 1.000 | 789.269 | 4.235 | .044 | .070 | 4.235 | .525 |
| surface * group | Sphericity Assumed | 122.958 | 1 | 122.958 | .660 | .420 | .012 | .660 | .126 |
|  | Greenhouse-Geisser | 122.958 | 1.000 | 122.958 | .660 | .420 | .012 | .660 | .126 |
|  | Huynh-Feldt | 122.958 | 1.000 | 122.958 | .660 | .420 | .012 | .660 | .126 |
|  | Lower-bound | 122.958 | 1.000 | 122.958 | .660 | .420 | .012 | .660 | .126 |
| Error(surface) | Sphericity Assumed | 10437.067 | 56 | 186.376 |  |  |  |  |  |
|  | Greenhouse-Geisser | 10437.067 | 56.000 | 186.376 |  |  |  |  |  |
|  | Huynh-Feldt | 10437.067 | 56.000 | 186.376 |  |  |  |  |  |
|  | Lower-bound | 10437.067 | 56.000 | 186.376 |  |  |  |  |  |
| a. Computed using alpha = .05 | | | | | | | | | |

| **Tests of Between-Subjects Effects** | | | | | | | | |
| --- | --- | --- | --- | --- | --- | --- | --- | --- |
| Measure: MEASURE_1 | | | | | | | | |
| Transformed Variable: Average | | | | | | | | |
| Source | Type III Sum of Squares | df | Mean Square | F | Sig. | Partial Eta Squared | Noncent. Parameter | Observed Power^a^ |
| Intercept | 16472.548 | 1 | 16472.548 | 93.575 | .000 | .626 | 93.575 | 1.000 |
| group | 134.191 | 1 | 134.191 | .762 | .386 | .013 | .762 | .138 |
| Error | 9858.023 | 56 | 176.036 |  |  |  |  |  |
| a. Computed using alpha = .05 | | | | | | | | |

| **Descriptive Statistics** | | | | |
| --- | --- | --- | --- | --- |
|  | group | Mean | Std. Deviation | N |
| levelTFx2 | pronated | 442.7667 | 162.98946 | 30 |
|  | healthy | 384.1034 | 193.50883 | 29 |
|  | Total | 413.9322 | 179.54088 | 59 |
| SandTFx2 | pronated | 483.6333 | 160.56140 | 30 |
|  | healthy | 432.1724 | 195.62283 | 29 |
|  | Total | 458.3390 | 178.99052 | 59 |

| **Tests of Within-Subjects Effects** | | | | | | | | | |
| --- | --- | --- | --- | --- | --- | --- | --- | --- | --- |
| Measure: MEASURE_1 | | | | | | | | | |
| Source | | Type III Sum of Squares | df | Mean Square | F | Sig. | Partial Eta Squared | Noncent. Parameter | Observed Power^a^ |
| surface | Sphericity Assumed | 7791.125 | 1 | 7791.125 | .395 | .532 | .007 | .395 | .095 |
|  | Greenhouse-Geisser | 7791.125 | 1.000 | 7791.125 | .395 | .532 | .007 | .395 | .095 |
|  | Huynh-Feldt | 7791.125 | 1.000 | 7791.125 | .395 | .532 | .007 | .395 | .095 |
|  | Lower-bound | 7791.125 | 1.000 | 7791.125 | .395 | .532 | .007 | .395 | .095 |
| surface * group | Sphericity Assumed | 627.278 | 1 | 627.278 | .032 | .859 | .001 | .032 | .054 |
|  | Greenhouse-Geisser | 627.278 | 1.000 | 627.278 | .032 | .859 | .001 | .032 | .054 |
|  | Huynh-Feldt | 627.278 | 1.000 | 627.278 | .032 | .859 | .001 | .032 | .054 |
|  | Lower-bound | 627.278 | 1.000 | 627.278 | .032 | .859 | .001 | .032 | .054 |
| Error(surface) | Sphericity Assumed | 1105420.988 | 56 | 19739.660 |  |  |  |  |  |
|  | Greenhouse-Geisser | 1105420.988 | 56.000 | 19739.660 |  |  |  |  |  |
|  | Huynh-Feldt | 1105420.988 | 56.000 | 19739.660 |  |  |  |  |  |
|  | Lower-bound | 1105420.988 | 56.000 | 19739.660 |  |  |  |  |  |
| a. Computed using alpha = .05 | | | | | | | | | |

| **Tests of Between-Subjects Effects** | | | | | | | | |
| --- | --- | --- | --- | --- | --- | --- | --- | --- |
| Measure: MEASURE_1 | | | | | | | | |
| Transformed Variable: Average | | | | | | | | |
| Source | Type III Sum of Squares | df | Mean Square | F | Sig. | Partial Eta Squared | Noncent. Parameter | Observed Power^a^ |
| Intercept | 1569321.434 | 1 | 1569321.434 | 35.062 | .000 | .385 | 35.062 | 1.000 |
| group | 114918.989 | 1 | 114918.989 | 2.568 | .115 | .044 | 2.568 | .350 |
| Error | 2506442.368 | 56 | 44757.899 |  |  |  |  |  |
| a. Computed using alpha = .05 | | | | | | | | |

| **Descriptive Statistics** | | | | |
| --- | --- | --- | --- | --- |
|  | group | Mean | Std. Deviation | N |
| levelTFy1 | pronated | 128.4667 | 57.95999 | 30 |
|  | healthy | 151.7586 | 66.33446 | 29 |
|  | Total | 139.9153 | 62.78433 | 59 |
| SandTFy1 | pronated | 126.4333 | 52.61092 | 30 |
|  | healthy | 160.6897 | 122.19338 | 29 |
|  | Total | 143.2712 | 94.28933 | 59 |

| **Tests of Within-Subjects Effects** | | | | | | | | | |
| --- | --- | --- | --- | --- | --- | --- | --- | --- | --- |
| Measure: MEASURE_1 | | | | | | | | | |
| Source | | Type III Sum of Squares | df | Mean Square | F | Sig. | Partial Eta Squared | Noncent. Parameter | Observed Power^a^ |
| surface | Sphericity Assumed | 13310.001 | 1 | 13310.001 | 2.356 | .130 | .040 | 2.356 | .326 |
|  | Greenhouse-Geisser | 13310.001 | 1.000 | 13310.001 | 2.356 | .130 | .040 | 2.356 | .326 |
|  | Huynh-Feldt | 13310.001 | 1.000 | 13310.001 | 2.356 | .130 | .040 | 2.356 | .326 |
|  | Lower-bound | 13310.001 | 1.000 | 13310.001 | 2.356 | .130 | .040 | 2.356 | .326 |
| surface * group | Sphericity Assumed | 5745.802 | 1 | 5745.802 | 1.017 | .318 | .018 | 1.017 | .168 |
|  | Greenhouse-Geisser | 5745.802 | 1.000 | 5745.802 | 1.017 | .318 | .018 | 1.017 | .168 |
|  | Huynh-Feldt | 5745.802 | 1.000 | 5745.802 | 1.017 | .318 | .018 | 1.017 | .168 |
|  | Lower-bound | 5745.802 | 1.000 | 5745.802 | 1.017 | .318 | .018 | 1.017 | .168 |
| Error(surface) | Sphericity Assumed | 316381.760 | 56 | 5649.674 |  |  |  |  |  |
|  | Greenhouse-Geisser | 316381.760 | 56.000 | 5649.674 |  |  |  |  |  |
|  | Huynh-Feldt | 316381.760 | 56.000 | 5649.674 |  |  |  |  |  |
|  | Lower-bound | 316381.760 | 56.000 | 5649.674 |  |  |  |  |  |
| a. Computed using alpha = .05 | | | | | | | | | |

| **Tests of Between-Subjects Effects** | | | | | | | | |
| --- | --- | --- | --- | --- | --- | --- | --- | --- |
| Measure: MEASURE_1 | | | | | | | | |
| Transformed Variable: Average | | | | | | | | |
| Source | Type III Sum of Squares | df | Mean Square | F | Sig. | Partial Eta Squared | Noncent. Parameter | Observed Power^a^ |
| Intercept | 76879.823 | 1 | 76879.823 | 12.175 | .001 | .179 | 12.175 | .929 |
| group | 3738.369 | 1 | 3738.369 | .592 | .445 | .010 | .592 | .118 |
| Error | 353611.680 | 56 | 6314.494 |  |  |  |  |  |
| a. Computed using alpha = .05 | | | | | | | | |

| **Descriptive Statistics** | | | | |
| --- | --- | --- | --- | --- |
|  | group | Mean | Std. Deviation | N |
| levelTFy2 | pronated | 608.4667 | 66.07557 | 30 |
|  | healthy | 591.6207 | 115.30222 | 29 |
|  | Total | 600.1864 | 93.13014 | 59 |
| SandTFy2 | pronated | 588.9333 | 114.50341 | 30 |
|  | healthy | 546.1724 | 165.36606 | 29 |
|  | Total | 567.9153 | 142.20363 | 59 |

| **Tests of Within-Subjects Effects** | | | | | | | | | |
| --- | --- | --- | --- | --- | --- | --- | --- | --- | --- |
| Measure: MEASURE_1 | | | | | | | | | |
| Source | | Type III Sum of Squares | df | Mean Square | F | Sig. | Partial Eta Squared | Noncent. Parameter | Observed Power^a^ |
| surface | Sphericity Assumed | 217.290 | 1 | 217.290 | .013 | .908 | .000 | .013 | .051 |
|  | Greenhouse-Geisser | 217.290 | 1.000 | 217.290 | .013 | .908 | .000 | .013 | .051 |
|  | Huynh-Feldt | 217.290 | 1.000 | 217.290 | .013 | .908 | .000 | .013 | .051 |
|  | Lower-bound | 217.290 | 1.000 | 217.290 | .013 | .908 | .000 | .013 | .051 |
| surface * group | Sphericity Assumed | 2202.712 | 1 | 2202.712 | .135 | .714 | .002 | .135 | .065 |
|  | Greenhouse-Geisser | 2202.712 | 1.000 | 2202.712 | .135 | .714 | .002 | .135 | .065 |
|  | Huynh-Feldt | 2202.712 | 1.000 | 2202.712 | .135 | .714 | .002 | .135 | .065 |
|  | Lower-bound | 2202.712 | 1.000 | 2202.712 | .135 | .714 | .002 | .135 | .065 |
| Error(surface) | Sphericity Assumed | 912163.706 | 56 | 16288.638 |  |  |  |  |  |
|  | Greenhouse-Geisser | 912163.706 | 56.000 | 16288.638 |  |  |  |  |  |
|  | Huynh-Feldt | 912163.706 | 56.000 | 16288.638 |  |  |  |  |  |
|  | Lower-bound | 912163.706 | 56.000 | 16288.638 |  |  |  |  |  |
| a. Computed using alpha = .05 | | | | | | | | | |

| **Tests of Between-Subjects Effects** | | | | | | | | |
| --- | --- | --- | --- | --- | --- | --- | --- | --- |
| Measure: MEASURE_1 | | | | | | | | |
| Transformed Variable: Average | | | | | | | | |
| Source | Type III Sum of Squares | df | Mean Square | F | Sig. | Partial Eta Squared | Noncent. Parameter | Observed Power^a^ |
| Intercept | 3355242.467 | 1 | 3355242.467 | 258.093 | .000 | .822 | 258.093 | 1.000 |
| group | 28879.139 | 1 | 28879.139 | 2.221 | .142 | .038 | 2.221 | .311 |
| Error | 728007.335 | 56 | 13000.131 |  |  |  |  |  |
| a. Computed using alpha = .05 | | | | | | | | |

| **Descriptive Statistics** | | | | |
| --- | --- | --- | --- | --- |
|  | group | Mean | Std. Deviation | N |
| levelTFZ1 | pronated | 170.5000 | 66.06410 | 30 |
|  | healthy | 174.0000 | 58.94610 | 29 |
|  | Total | 172.2203 | 62.15111 | 59 |
| SandTFz1 | pronated | 174.6333 | 71.02524 | 30 |
|  | healthy | 196.3103 | 122.73552 | 29 |
|  | Total | 185.2881 | 99.56925 | 59 |

| **Tests of Within-Subjects Effects** | | | | | | | | | |
| --- | --- | --- | --- | --- | --- | --- | --- | --- | --- |
| Measure: MEASURE_1 | | | | | | | | | |
| Source | | Type III Sum of Squares | df | Mean Square | F | Sig. | Partial Eta Squared | Noncent. Parameter | Observed Power^a^ |
| surface | Sphericity Assumed | 9.864 | 1 | 9.864 | .002 | .965 | .000 | .002 | .050 |
|  | Greenhouse-Geisser | 9.864 | 1.000 | 9.864 | .002 | .965 | .000 | .002 | .050 |
|  | Huynh-Feldt | 9.864 | 1.000 | 9.864 | .002 | .965 | .000 | .002 | .050 |
|  | Lower-bound | 9.864 | 1.000 | 9.864 | .002 | .965 | .000 | .002 | .050 |
| surface * group | Sphericity Assumed | 1141.877 | 1 | 1141.877 | .222 | .640 | .004 | .222 | .075 |
|  | Greenhouse-Geisser | 1141.877 | 1.000 | 1141.877 | .222 | .640 | .004 | .222 | .075 |
|  | Huynh-Feldt | 1141.877 | 1.000 | 1141.877 | .222 | .640 | .004 | .222 | .075 |
|  | Lower-bound | 1141.877 | 1.000 | 1141.877 | .222 | .640 | .004 | .222 | .075 |
| Error(surface) | Sphericity Assumed | 288638.552 | 56 | 5154.260 |  |  |  |  |  |
|  | Greenhouse-Geisser | 288638.552 | 56.000 | 5154.260 |  |  |  |  |  |
|  | Huynh-Feldt | 288638.552 | 56.000 | 5154.260 |  |  |  |  |  |
|  | Lower-bound | 288638.552 | 56.000 | 5154.260 |  |  |  |  |  |
| a. Computed using alpha = .05 | | | | | | | | | |

| **Tests of Between-Subjects Effects** | | | | | | | | |
| --- | --- | --- | --- | --- | --- | --- | --- | --- |
| Measure: MEASURE_1 | | | | | | | | |
| Transformed Variable: Average | | | | | | | | |
| Source | Type III Sum of Squares | df | Mean Square | F | Sig. | Partial Eta Squared | Noncent. Parameter | Observed Power^a^ |
| Intercept | 81957.972 | 1 | 81957.972 | 11.201 | .001 | .167 | 11.201 | .908 |
| group | 4568.085 | 1 | 4568.085 | .624 | .433 | .011 | .624 | .121 |
| Error | 409739.889 | 56 | 7316.784 |  |  |  |  |  |
| a. Computed using alpha = .05 | | | | | | | | |

| **Descriptive Statistics** | | | | |
| --- | --- | --- | --- | --- |
|  | group | Mean | Std. Deviation | N |
| levelTFZ2 | pronated | 329.2667 | 52.13704 | 30 |
|  | healthy | 341.6552 | 63.06532 | 29 |
|  | Total | 335.3559 | 57.60381 | 59 |
| SandTFz2 | pronated | 353.1000 | 62.59027 | 30 |
|  | healthy | 370.4828 | 171.70440 | 29 |
|  | Total | 361.6441 | 127.54793 | 59 |

| **Tests of Within-Subjects Effects** | | | | | | | | | |
| --- | --- | --- | --- | --- | --- | --- | --- | --- | --- |
| Measure: MEASURE_1 | | | | | | | | | |
| Source | | Type III Sum of Squares | df | Mean Square | F | Sig. | Partial Eta Squared | Noncent. Parameter | Observed Power^a^ |
| surface | Sphericity Assumed | 2151.117 | 1 | 2151.117 | .251 | .619 | .004 | .251 | .078 |
|  | Greenhouse-Geisser | 2151.117 | 1.000 | 2151.117 | .251 | .619 | .004 | .251 | .078 |
|  | Huynh-Feldt | 2151.117 | 1.000 | 2151.117 | .251 | .619 | .004 | .251 | .078 |
|  | Lower-bound | 2151.117 | 1.000 | 2151.117 | .251 | .619 | .004 | .251 | .078 |
| surface * group | Sphericity Assumed | 743.197 | 1 | 743.197 | .087 | .770 | .002 | .087 | .060 |
|  | Greenhouse-Geisser | 743.197 | 1.000 | 743.197 | .087 | .770 | .002 | .087 | .060 |
|  | Huynh-Feldt | 743.197 | 1.000 | 743.197 | .087 | .770 | .002 | .087 | .060 |
|  | Lower-bound | 743.197 | 1.000 | 743.197 | .087 | .770 | .002 | .087 | .060 |
| Error(surface) | Sphericity Assumed | 480596.314 | 56 | 8582.077 |  |  |  |  |  |
|  | Greenhouse-Geisser | 480596.314 | 56.000 | 8582.077 |  |  |  |  |  |
|  | Huynh-Feldt | 480596.314 | 56.000 | 8582.077 |  |  |  |  |  |
|  | Lower-bound | 480596.314 | 56.000 | 8582.077 |  |  |  |  |  |
| a. Computed using alpha = .05 | | | | | | | | | |

| **Tests of Between-Subjects Effects** | | | | | | | | |
| --- | --- | --- | --- | --- | --- | --- | --- | --- |
| Measure: MEASURE_1 | | | | | | | | |
| Transformed Variable: Average | | | | | | | | |
| Source | Type III Sum of Squares | df | Mean Square | F | Sig. | Partial Eta Squared | Noncent. Parameter | Observed Power^a^ |
| Intercept | 813572.086 | 1 | 813572.086 | 77.833 | .000 | .582 | 77.833 | 1.000 |
| group | 684.332 | 1 | 684.332 | .065 | .799 | .001 | .065 | .057 |
| Error | 585353.906 | 56 | 10452.748 |  |  |  |  |  |
| a. Computed using alpha = .05 | | | | | | | | |

| **Descriptive Statistics** | | | | |
| --- | --- | --- | --- | --- |
|  | group | Mean | Std. Deviation | N |
| levelTFz3 | pronated | 536.0000 | 63.39966 | 30 |
|  | healthy | 538.5862 | 74.38919 | 29 |
|  | Total | 537.2712 | 68.43190 | 59 |
| SandTFz3 | pronated | 550.4333 | 46.59487 | 30 |
|  | healthy | 554.8966 | 179.43709 | 29 |
|  | Total | 552.6271 | 128.97405 | 59 |

| **Tests of Within-Subjects Effects** | | | | | | | | | |
| --- | --- | --- | --- | --- | --- | --- | --- | --- | --- |
| Measure: MEASURE_1 | | | | | | | | | |
| Source | | Type III Sum of Squares | df | Mean Square | F | Sig. | Partial Eta Squared | Noncent. Parameter | Observed Power^a^ |
| Surface | Sphericity Assumed | 835.302 | 1 | 835.302 | .084 | .773 | .001 | .084 | .059 |
|  | Greenhouse-Geisser | 835.302 | 1.000 | 835.302 | .084 | .773 | .001 | .084 | .059 |
|  | Huynh-Feldt | 835.302 | 1.000 | 835.302 | .084 | .773 | .001 | .084 | .059 |
|  | Lower-bound | 835.302 | 1.000 | 835.302 | .084 | .773 | .001 | .084 | .059 |
| surface * group | Sphericity Assumed | 41.409 | 1 | 41.409 | .004 | .949 | .000 | .004 | .050 |
|  | Greenhouse-Geisser | 41.409 | 1.000 | 41.409 | .004 | .949 | .000 | .004 | .050 |
|  | Huynh-Feldt | 41.409 | 1.000 | 41.409 | .004 | .949 | .000 | .004 | .050 |
|  | Lower-bound | 41.409 | 1.000 | 41.409 | .004 | .949 | .000 | .004 | .050 |
| Error(surface) | Sphericity Assumed | 558973.409 | 56 | 9981.668 |  |  |  |  |  |
|  | Greenhouse-Geisser | 558973.409 | 56.000 | 9981.668 |  |  |  |  |  |
|  | Huynh-Feldt | 558973.409 | 56.000 | 9981.668 |  |  |  |  |  |
|  | Lower-bound | 558973.409 | 56.000 | 9981.668 |  |  |  |  |  |
| a. Computed using alpha = .05 | | | | | | | | | |

| **Tests of Between-Subjects Effects** | | | | | | | | |
| --- | --- | --- | --- | --- | --- | --- | --- | --- |
| Measure: MEASURE_1 | | | | | | | | |
| Transformed Variable: Average | | | | | | | | |
| Source | Type III Sum of Squares | df | Mean Square | F | Sig. | Partial Eta Squared | Noncent. Parameter | Observed Power^a^ |
| Intercept | 2456284.899 | 1 | 2456284.899 | 216.053 | .000 | .794 | 216.053 | 1.000 |
| group | 4624.595 | 1 | 4624.595 | .407 | .526 | .007 | .407 | .096 |
| Error | 636659.295 | 56 | 11368.916 |  |  |  |  |  |
| a. Computed using alpha = .05 | | | | | | | | |

| **Descriptive Statistics** | | | | |
| --- | --- | --- | --- | --- |
|  | group | Mean | Std. Deviation | N |
| LevelLRBFP | pronated | 9.2614 | 5.02388 | 30 |
|  | healthy | 13.4851 | 7.32040 | 29 |
|  | Total | 11.3375 | 6.55936 | 59 |
| SandLRBFP | pronated | 10.9565 | 9.47640 | 30 |
|  | healthy | 15.5567 | 10.06100 | 29 |
|  | Total | 13.2177 | 9.95730 | 59 |

| **Tests of Within-Subjects Effects** | | | | | | | | | |
| --- | --- | --- | --- | --- | --- | --- | --- | --- | --- |
| Measure: MEASURE_1 | | | | | | | | | |
| Source | | Type III Sum of Squares | df | Mean Square | F | Sig. | Partial Eta Squared | Noncent. Parameter | Observed Power^a^ |
| surface | Sphericity Assumed | .047 | 1 | .047 | .001 | .974 | .000 | .001 | .050 |
|  | Greenhouse-Geisser | .047 | 1.000 | .047 | .001 | .974 | .000 | .001 | .050 |
|  | Huynh-Feldt | .047 | 1.000 | .047 | .001 | .974 | .000 | .001 | .050 |
|  | Lower-bound | .047 | 1.000 | .047 | .001 | .974 | .000 | .001 | .050 |
| surface * group | Sphericity Assumed | .274 | 1 | .274 | .006 | .938 | .000 | .006 | .051 |
|  | Greenhouse-Geisser | .274 | 1.000 | .274 | .006 | .938 | .000 | .006 | .051 |
|  | Huynh-Feldt | .274 | 1.000 | .274 | .006 | .938 | .000 | .006 | .051 |
|  | Lower-bound | .274 | 1.000 | .274 | .006 | .938 | .000 | .006 | .051 |
| Error(surface) | Sphericity Assumed | 2478.348 | 56 | 44.256 |  |  |  |  |  |
|  | Greenhouse-Geisser | 2478.348 | 56.000 | 44.256 |  |  |  |  |  |
|  | Huynh-Feldt | 2478.348 | 56.000 | 44.256 |  |  |  |  |  |
|  | Lower-bound | 2478.348 | 56.000 | 44.256 |  |  |  |  |  |
| a. Computed using alpha = .05 | | | | | | | | | |

| **Tests of Between-Subjects Effects** | | | | | | | | |
| --- | --- | --- | --- | --- | --- | --- | --- | --- |
| Measure: MEASURE_1 | | | | | | | | |
| Transformed Variable: Average | | | | | | | | |
| Source | Type III Sum of Squares | df | Mean Square | F | Sig. | Partial Eta Squared | Noncent. Parameter | Observed Power^a^ |
| Intercept | 821.085 | 1 | 821.085 | 9.112 | .004 | .140 | 9.112 | .843 |
| group | 280.708 | 1 | 280.708 | 3.115 | .083 | .053 | 3.115 | .411 |
| Error | 5045.929 | 56 | 90.106 |  |  |  |  |  |
| a. Computed using alpha = .05 | | | | | | | | |

| **Descriptive Statistics** | | | | |
| --- | --- | --- | --- | --- |
|  | group | Mean | Std. Deviation | N |
| LevelLRGasMP | pronated | 12.1131 | 14.31691 | 30 |
|  | healthy | 12.6624 | 11.20372 | 29 |
|  | Total | 12.3831 | 12.77345 | 59 |
| SandLRGasMP | pronated | 7.7234 | 8.64262 | 30 |
|  | healthy | 11.8667 | 12.15657 | 29 |
|  | Total | 9.7599 | 10.63273 | 59 |

| **Tests of Within-Subjects Effects** | | | | | | | | | |
| --- | --- | --- | --- | --- | --- | --- | --- | --- | --- |
| Measure: MEASURE_1 | | | | | | | | | |
| Source | | Type III Sum of Squares | df | Mean Square | F | Sig. | Partial Eta Squared | Noncent. Parameter | Observed Power^a^ |
| surface | Sphericity Assumed | 53.963 | 1 | 53.963 | .902 | .346 | .016 | .902 | .154 |
|  | Greenhouse-Geisser | 53.963 | 1.000 | 53.963 | .902 | .346 | .016 | .902 | .154 |
|  | Huynh-Feldt | 53.963 | 1.000 | 53.963 | .902 | .346 | .016 | .902 | .154 |
|  | Lower-bound | 53.963 | 1.000 | 53.963 | .902 | .346 | .016 | .902 | .154 |
| surface * group | Sphericity Assumed | 55.179 | 1 | 55.179 | .922 | .341 | .016 | .922 | .157 |
|  | Greenhouse-Geisser | 55.179 | 1.000 | 55.179 | .922 | .341 | .016 | .922 | .157 |
|  | Huynh-Feldt | 55.179 | 1.000 | 55.179 | .922 | .341 | .016 | .922 | .157 |
|  | Lower-bound | 55.179 | 1.000 | 55.179 | .922 | .341 | .016 | .922 | .157 |
| Error(surface) | Sphericity Assumed | 3351.937 | 56 | 59.856 |  |  |  |  |  |
|  | Greenhouse-Geisser | 3351.937 | 56.000 | 59.856 |  |  |  |  |  |
|  | Huynh-Feldt | 3351.937 | 56.000 | 59.856 |  |  |  |  |  |
|  | Lower-bound | 3351.937 | 56.000 | 59.856 |  |  |  |  |  |
| a. Computed using alpha = .05 | | | | | | | | | |

| **Tests of Between-Subjects Effects** | | | | | | | | |
| --- | --- | --- | --- | --- | --- | --- | --- | --- |
| Measure: MEASURE_1 | | | | | | | | |
| Transformed Variable: Average | | | | | | | | |
| Source | Type III Sum of Squares | df | Mean Square | F | Sig. | Partial Eta Squared | Noncent. Parameter | Observed Power^a^ |
| Intercept | 640.615 | 1 | 640.615 | 4.320 | .042 | .072 | 4.320 | .533 |
| group | 244.976 | 1 | 244.976 | 1.652 | .204 | .029 | 1.652 | .244 |
| Error | 8303.737 | 56 | 148.281 |  |  |  |  |  |
| a. Computed using alpha = .05 | | | | | | | | |

| **Descriptive Statistics** | | | | |
| --- | --- | --- | --- | --- |
|  | group | Mean | Std. Deviation | N |
| LevelLRGluMP | pronated | 19.4546 | 15.29359 | 30 |
|  | healthy | 25.1495 | 22.36562 | 29 |
|  | Total | 22.2538 | 19.14885 | 59 |
| SandLRGluMP | pronated | 15.6886 | 9.62875 | 30 |
|  | healthy | 21.3851 | 13.10681 | 29 |
|  | Total | 18.4886 | 11.72771 | 59 |

| **Tests of Within-Subjects Effects** | | | | | | | | | |
| --- | --- | --- | --- | --- | --- | --- | --- | --- | --- |
| Measure: MEASURE_1 | | | | | | | | | |
| Source | | Type III Sum of Squares | df | Mean Square | F | Sig. | Partial Eta Squared | Noncent. Parameter | Observed Power^a^ |
| surface | Sphericity Assumed | .178 | 1 | .178 | .001 | .976 | .000 | .001 | .050 |
|  | Greenhouse-Geisser | .178 | 1.000 | .178 | .001 | .976 | .000 | .001 | .050 |
|  | Huynh-Feldt | .178 | 1.000 | .178 | .001 | .976 | .000 | .001 | .050 |
|  | Lower-bound | .178 | 1.000 | .178 | .001 | .976 | .000 | .001 | .050 |
| surface * group | Sphericity Assumed | 6.348 | 1 | 6.348 | .031 | .860 | .001 | .031 | .053 |
|  | Greenhouse-Geisser | 6.348 | 1.000 | 6.348 | .031 | .860 | .001 | .031 | .053 |
|  | Huynh-Feldt | 6.348 | 1.000 | 6.348 | .031 | .860 | .001 | .031 | .053 |
|  | Lower-bound | 6.348 | 1.000 | 6.348 | .031 | .860 | .001 | .031 | .053 |
| Error(surface) | Sphericity Assumed | 11328.378 | 56 | 202.292 |  |  |  |  |  |
|  | Greenhouse-Geisser | 11328.378 | 56.000 | 202.292 |  |  |  |  |  |
|  | Huynh-Feldt | 11328.378 | 56.000 | 202.292 |  |  |  |  |  |
|  | Lower-bound | 11328.378 | 56.000 | 202.292 |  |  |  |  |  |
| a. Computed using alpha = .05 | | | | | | | | | |

| **Tests of Between-Subjects Effects** | | | | | | | | |
| --- | --- | --- | --- | --- | --- | --- | --- | --- |
| Measure: MEASURE_1 | | | | | | | | |
| Transformed Variable: Average | | | | | | | | |
| Source | Type III Sum of Squares | df | Mean Square | F | Sig. | Partial Eta Squared | Noncent. Parameter | Observed Power^a^ |
| Intercept | 1181.086 | 1 | 1181.086 | 4.177 | .046 | .069 | 4.177 | .519 |
| group | 195.149 | 1 | 195.149 | .690 | .410 | .012 | .690 | .129 |
| Error | 15833.117 | 56 | 282.734 |  |  |  |  |  |
| a. Computed using alpha = .05 | | | | | | | | |

| **Descriptive Statistics** | | | | |
| --- | --- | --- | --- | --- |
|  | group | Mean | Std. Deviation | N |
| LevelLRRFP | pronated | 17.6550 | 9.91177 | 30 |
|  | healthy | 20.2725 | 14.49457 | 29 |
|  | Total | 18.9416 | 12.34048 | 59 |
| SandLRRFP | pronated | 16.9339 | 6.95416 | 30 |
|  | healthy | 20.7432 | 14.26588 | 29 |
|  | Total | 18.8063 | 11.23023 | 59 |

| **Tests of Within-Subjects Effects** | | | | | | | | | |
| --- | --- | --- | --- | --- | --- | --- | --- | --- | --- |
| Measure: MEASURE_1 | | | | | | | | | |
| Source | | Type III Sum of Squares | df | Mean Square | F | Sig. | Partial Eta Squared | Noncent. Parameter | Observed Power^a^ |
| surface | Sphericity Assumed | 4.661 | 1 | 4.661 | .263 | .610 | .005 | .263 | .080 |
|  | Greenhouse-Geisser | 4.661 | 1.000 | 4.661 | .263 | .610 | .005 | .263 | .080 |
|  | Huynh-Feldt | 4.661 | 1.000 | 4.661 | .263 | .610 | .005 | .263 | .080 |
|  | Lower-bound | 4.661 | 1.000 | 4.661 | .263 | .610 | .005 | .263 | .080 |
| surface * group | Sphericity Assumed | 15.839 | 1 | 15.839 | .893 | .349 | .016 | .893 | .153 |
|  | Greenhouse-Geisser | 15.839 | 1.000 | 15.839 | .893 | .349 | .016 | .893 | .153 |
|  | Huynh-Feldt | 15.839 | 1.000 | 15.839 | .893 | .349 | .016 | .893 | .153 |
|  | Lower-bound | 15.839 | 1.000 | 15.839 | .893 | .349 | .016 | .893 | .153 |
| Error(surface) | Sphericity Assumed | 993.394 | 56 | 17.739 |  |  |  |  |  |
|  | Greenhouse-Geisser | 993.394 | 56.000 | 17.739 |  |  |  |  |  |
|  | Huynh-Feldt | 993.394 | 56.000 | 17.739 |  |  |  |  |  |
|  | Lower-bound | 993.394 | 56.000 | 17.739 |  |  |  |  |  |
| a. Computed using alpha = .05 | | | | | | | | | |

| **Tests of Between-Subjects Effects** | | | | | | | | |
| --- | --- | --- | --- | --- | --- | --- | --- | --- |
| Measure: MEASURE_1 | | | | | | | | |
| Transformed Variable: Average | | | | | | | | |
| Source | Type III Sum of Squares | df | Mean Square | F | Sig. | Partial Eta Squared | Noncent. Parameter | Observed Power^a^ |
| Intercept | 399.893 | 1 | 399.893 | 1.724 | .195 | .030 | 1.724 | .252 |
| group | 5.988 | 1 | 5.988 | .026 | .873 | .000 | .026 | .053 |
| Error | 12988.161 | 56 | 231.931 |  |  |  |  |  |
| a. Computed using alpha = .05 | | | | | | | | |

| **Descriptive Statistics** | | | | |
| --- | --- | --- | --- | --- |
|  | group | Mean | Std. Deviation | N |
| LevelLRSTP | pronated | 15.0012 | 20.85369 | 30 |
|  | healthy | 14.6658 | 10.83317 | 29 |
|  | Total | 14.8363 | 16.55663 | 59 |
| SandLRSTP | pronated | 11.0509 | 9.90457 | 30 |
|  | healthy | 14.4418 | 14.65116 | 29 |
|  | Total | 12.7176 | 12.47401 | 59 |

| **Tests of Within-Subjects Effects** | | | | | | | | | |
| --- | --- | --- | --- | --- | --- | --- | --- | --- | --- |
| Measure: MEASURE_1 | | | | | | | | | |
| Source | | Type III Sum of Squares | df | Mean Square | F | Sig. | Partial Eta Squared | Noncent. Parameter | Observed Power^a^ |
| surface | Sphericity Assumed | 68.071 | 1 | 68.071 | .504 | .481 | .009 | .504 | .107 |
|  | Greenhouse-Geisser | 68.071 | 1.000 | 68.071 | .504 | .481 | .009 | .504 | .107 |
|  | Huynh-Feldt | 68.071 | 1.000 | 68.071 | .504 | .481 | .009 | .504 | .107 |
|  | Lower-bound | 68.071 | 1.000 | 68.071 | .504 | .481 | .009 | .504 | .107 |
| surface * group | Sphericity Assumed | 48.831 | 1 | 48.831 | .361 | .550 | .006 | .361 | .091 |
|  | Greenhouse-Geisser | 48.831 | 1.000 | 48.831 | .361 | .550 | .006 | .361 | .091 |
|  | Huynh-Feldt | 48.831 | 1.000 | 48.831 | .361 | .550 | .006 | .361 | .091 |
|  | Lower-bound | 48.831 | 1.000 | 48.831 | .361 | .550 | .006 | .361 | .091 |
| Error(surface) | Sphericity Assumed | 7565.604 | 56 | 135.100 |  |  |  |  |  |
|  | Greenhouse-Geisser | 7565.604 | 56.000 | 135.100 |  |  |  |  |  |
|  | Huynh-Feldt | 7565.604 | 56.000 | 135.100 |  |  |  |  |  |
|  | Lower-bound | 7565.604 | 56.000 | 135.100 |  |  |  |  |  |
| a. Computed using alpha = .05 | | | | | | | | | |

| **Tests of Between-Subjects Effects** | | | | | | | | |
| --- | --- | --- | --- | --- | --- | --- | --- | --- |
| Measure: MEASURE_1 | | | | | | | | |
| Transformed Variable: Average | | | | | | | | |
| Source | Type III Sum of Squares | df | Mean Square | F | Sig. | Partial Eta Squared | Noncent. Parameter | Observed Power^a^ |
| Intercept | 3016.978 | 1 | 3016.978 | 9.914 | .003 | .150 | 9.914 | .872 |
| group | 147.495 | 1 | 147.495 | .485 | .489 | .009 | .485 | .105 |
| Error | 17041.966 | 56 | 304.321 |  |  |  |  |  |
| a. Computed using alpha = .05 | | | | | | | | |

| **Descriptive Statistics** | | | | |
| --- | --- | --- | --- | --- |
|  | group | Mean | Std. Deviation | N |
| LevelLRtibialisP | pronated | 23.0621 | 12.37417 | 30 |
|  | healthy | 24.9437 | 13.76630 | 29 |
|  | Total | 23.9869 | 12.99801 | 59 |
| SandLRTAP | pronated | 24.9083 | 11.76670 | 30 |
|  | healthy | 25.2255 | 16.08632 | 29 |
|  | Total | 25.0642 | 13.93473 | 59 |

| **Tests of Within-Subjects Effects** | | | | | | | | | |
| --- | --- | --- | --- | --- | --- | --- | --- | --- | --- |
| Measure: MEASURE_1 | | | | | | | | | |
| Source | | Type III Sum of Squares | df | Mean Square | F | Sig. | Partial Eta Squared | Noncent. Parameter | Observed Power^a^ |
| surface | Sphericity Assumed | 10.009 | 1 | 10.009 | .123 | .728 | .002 | .123 | .064 |
|  | Greenhouse-Geisser | 10.009 | 1.000 | 10.009 | .123 | .728 | .002 | .123 | .064 |
|  | Huynh-Feldt | 10.009 | 1.000 | 10.009 | .123 | .728 | .002 | .123 | .064 |
|  | Lower-bound | 10.009 | 1.000 | 10.009 | .123 | .728 | .002 | .123 | .064 |
| surface * group | Sphericity Assumed | 10.253 | 1 | 10.253 | .126 | .724 | .002 | .126 | .064 |
|  | Greenhouse-Geisser | 10.253 | 1.000 | 10.253 | .126 | .724 | .002 | .126 | .064 |
|  | Huynh-Feldt | 10.253 | 1.000 | 10.253 | .126 | .724 | .002 | .126 | .064 |
|  | Lower-bound | 10.253 | 1.000 | 10.253 | .126 | .724 | .002 | .126 | .064 |
| Error(surface) | Sphericity Assumed | 4570.814 | 56 | 81.622 |  |  |  |  |  |
|  | Greenhouse-Geisser | 4570.814 | 56.000 | 81.622 |  |  |  |  |  |
|  | Huynh-Feldt | 4570.814 | 56.000 | 81.622 |  |  |  |  |  |
|  | Lower-bound | 4570.814 | 56.000 | 81.622 |  |  |  |  |  |
| a. Computed using alpha = .05 | | | | | | | | | |

| **Tests of Between-Subjects Effects** | | | | | | | | |
| --- | --- | --- | --- | --- | --- | --- | --- | --- |
| Measure: MEASURE_1 | | | | | | | | |
| Transformed Variable: Average | | | | | | | | |
| Source | Type III Sum of Squares | df | Mean Square | F | Sig. | Partial Eta Squared | Noncent. Parameter | Observed Power^a^ |
| Intercept | 801.962 | 1 | 801.962 | 3.303 | .074 | .056 | 3.303 | .431 |
| group | 296.800 | 1 | 296.800 | 1.223 | .274 | .021 | 1.223 | .192 |
| Error | 13595.551 | 56 | 242.778 |  |  |  |  |  |
| a. Computed using alpha = .05 | | | | | | | | |

| **Descriptive Statistics** | | | | |
| --- | --- | --- | --- | --- |
|  | group | Mean | Std. Deviation | N |
| LevelLRVLP | pronated | 21.2665 | 14.53721 | 30 |
|  | healthy | 17.2841 | 15.19713 | 29 |
|  | Total | 19.3091 | 14.87252 | 59 |
| SandLRVLP | pronated | 21.0693 | 16.51846 | 30 |
|  | healthy | 16.3481 | 14.25179 | 29 |
|  | Total | 18.7487 | 15.49681 | 59 |

| **Tests of Within-Subjects Effects** | | | | | | | | | |
| --- | --- | --- | --- | --- | --- | --- | --- | --- | --- |
| Measure: MEASURE_1 | | | | | | | | | |
| Source | | Type III Sum of Squares | df | Mean Square | F | Sig. | Partial Eta Squared | Noncent. Parameter | Observed Power^a^ |
| surface | Sphericity Assumed | 188.490 | 1 | 188.490 | 1.929 | .170 | .033 | 1.929 | .276 |
|  | Greenhouse-Geisser | 188.490 | 1.000 | 188.490 | 1.929 | .170 | .033 | 1.929 | .276 |
|  | Huynh-Feldt | 188.490 | 1.000 | 188.490 | 1.929 | .170 | .033 | 1.929 | .276 |
|  | Lower-bound | 188.490 | 1.000 | 188.490 | 1.929 | .170 | .033 | 1.929 | .276 |
| surface * group | Sphericity Assumed | 56.503 | 1 | 56.503 | .578 | .450 | .010 | .578 | .116 |
|  | Greenhouse-Geisser | 56.503 | 1.000 | 56.503 | .578 | .450 | .010 | .578 | .116 |
|  | Huynh-Feldt | 56.503 | 1.000 | 56.503 | .578 | .450 | .010 | .578 | .116 |
|  | Lower-bound | 56.503 | 1.000 | 56.503 | .578 | .450 | .010 | .578 | .116 |
| Error(surface) | Sphericity Assumed | 5471.662 | 56 | 97.708 |  |  |  |  |  |
|  | Greenhouse-Geisser | 5471.662 | 56.000 | 97.708 |  |  |  |  |  |
|  | Huynh-Feldt | 5471.662 | 56.000 | 97.708 |  |  |  |  |  |
|  | Lower-bound | 5471.662 | 56.000 | 97.708 |  |  |  |  |  |
| a. Computed using alpha = .05 | | | | | | | | | |

| **Tests of Between-Subjects Effects** | | | | | | | | |
| --- | --- | --- | --- | --- | --- | --- | --- | --- |
| Measure: MEASURE_1 | | | | | | | | |
| Transformed Variable: Average | | | | | | | | |
| Source | Type III Sum of Squares | df | Mean Square | F | Sig. | Partial Eta Squared | Noncent. Parameter | Observed Power^a^ |
| Intercept | 1703.582 | 1 | 1703.582 | 4.745 | .034 | .078 | 4.745 | .572 |
| group | 918.359 | 1 | 918.359 | 2.558 | .115 | .044 | 2.558 | .349 |
| Error | 20103.631 | 56 | 358.993 |  |  |  |  |  |
| a. Computed using alpha = .05 | | | | | | | | |

| **Descriptive Statistics** | | | | |
| --- | --- | --- | --- | --- |
|  | group | Mean | Std. Deviation | N |
| LevelLRVMP | pronated | 20.7856 | 13.52287 | 30 |
|  | healthy | 22.3687 | 17.67308 | 29 |
|  | Total | 21.5637 | 15.58380 | 59 |
| SandLRVMP | pronated | 16.3632 | 12.46169 | 30 |
|  | healthy | 27.5825 | 21.46133 | 29 |
|  | Total | 21.8777 | 18.22091 | 59 |

| **Tests of Within-Subjects Effects** | | | | | | | | | |
| --- | --- | --- | --- | --- | --- | --- | --- | --- | --- |
| Measure: MEASURE_1 | | | | | | | | | |
| Source | | Type III Sum of Squares | df | Mean Square | F | Sig. | Partial Eta Squared | Noncent. Parameter | Observed Power^a^ |
| surface | Sphericity Assumed | 44.206 | 1 | 44.206 | .223 | .639 | .004 | .223 | .075 |
|  | Greenhouse-Geisser | 44.206 | 1.000 | 44.206 | .223 | .639 | .004 | .223 | .075 |
|  | Huynh-Feldt | 44.206 | 1.000 | 44.206 | .223 | .639 | .004 | .223 | .075 |
|  | Lower-bound | 44.206 | 1.000 | 44.206 | .223 | .639 | .004 | .223 | .075 |
| surface * group | Sphericity Assumed | 418.014 | 1 | 418.014 | 2.109 | .152 | .036 | 2.109 | .297 |
|  | Greenhouse-Geisser | 418.014 | 1.000 | 418.014 | 2.109 | .152 | .036 | 2.109 | .297 |
|  | Huynh-Feldt | 418.014 | 1.000 | 418.014 | 2.109 | .152 | .036 | 2.109 | .297 |
|  | Lower-bound | 418.014 | 1.000 | 418.014 | 2.109 | .152 | .036 | 2.109 | .297 |
| Error(surface) | Sphericity Assumed | 11102.010 | 56 | 198.250 |  |  |  |  |  |
|  | Greenhouse-Geisser | 11102.010 | 56.000 | 198.250 |  |  |  |  |  |
|  | Huynh-Feldt | 11102.010 | 56.000 | 198.250 |  |  |  |  |  |
|  | Lower-bound | 11102.010 | 56.000 | 198.250 |  |  |  |  |  |
| a. Computed using alpha = .05 | | | | | | | | | |

| **Tests of Between-Subjects Effects** | | | | | | | | |
| --- | --- | --- | --- | --- | --- | --- | --- | --- |
| Measure: MEASURE_1 | | | | | | | | |
| Transformed Variable: Average | | | | | | | | |
| Source | Type III Sum of Squares | df | Mean Square | F | Sig. | Partial Eta Squared | Noncent. Parameter | Observed Power^a^ |
| Intercept | 317.835 | 1 | 317.835 | 1.031 | .314 | .018 | 1.031 | .170 |
| group | 66.154 | 1 | 66.154 | .215 | .645 | .004 | .215 | .074 |
| Error | 17270.104 | 56 | 308.395 |  |  |  |  |  |
| a. Computed using alpha = .05 | | | | | | | | |

| **Descriptive Statistics** | | | | |
| --- | --- | --- | --- | --- |
|  | group | Mean | Std. Deviation | N |
| LevelMidsBFP | pronated | 6.5497 | 7.04568 | 30 |
|  | healthy | 8.6259 | 6.57858 | 29 |
|  | Total | 7.5702 | 6.84174 | 59 |
| SandMidBFP | pronated | 7.7782 | 8.66746 | 30 |
|  | healthy | 12.8276 | 15.02637 | 29 |
|  | Total | 10.2601 | 12.37124 | 59 |

| Source | | Type III Sum of Squares | df | Mean Square | F | Sig. | Partial Eta Squared | Noncent. Parameter |
| --- | --- | --- | --- | --- | --- | --- | --- | --- |
| surface | Sphericity Assumed | .004 | 1 | .004 | .000 | .993 | .000 | .000 |
|  | Greenhouse-Geisser | .004 | 1.000 | .004 | .000 | .993 | .000 | .000 |
|  | Huynh-Feldt | .004 | 1.000 | .004 | .000 | .993 | .000 | .000 |
|  | Lower-bound | .004 | 1.000 | .004 | .000 | .993 | .000 | .000 |
| surface * group | Sphericity Assumed | 28.428 | 1 | 28.428 | .497 | .484 | .009 | .497 |
|  | Greenhouse-Geisser | 28.428 | 1.000 | 28.428 | .497 | .484 | .009 | .497 |
|  | Huynh-Feldt | 28.428 | 1.000 | 28.428 | .497 | .484 | .009 | .497 |
|  | Lower-bound | 28.428 | 1.000 | 28.428 | .497 | .484 | .009 | .497 |
| Error(surface) | Sphericity Assumed | 3204.236 | 56 | 57.219 |  |  |  |  |
|  | Greenhouse-Geisser | 3204.236 | 56.000 | 57.219 |  |  |  |  |
|  | Huynh-Feldt | 3204.236 | 56.000 | 57.219 |  |  |  |  |
|  | Lower-bound | 3204.236 | 56.000 | 57.219 |  |  |  |  |

| **Tests of Between-Subjects Effects** | | | | | | | | |
| --- | --- | --- | --- | --- | --- | --- | --- | --- |
| Measure: MEASURE_1 | | | | | | | | |
| Transformed Variable: Average | | | | | | | | |
| Source | Type III Sum of Squares | df | Mean Square | F | Sig. | Partial Eta Squared | Noncent. Parameter | Observed Power^a^ |
| Intercept | 19.179 | 1 | 19.179 | .148 | .702 | .003 | .148 | .067 |
| group | 43.809 | 1 | 43.809 | .338 | .564 | .006 | .338 | .088 |
| Error | 7268.202 | 56 | 129.789 |  |  |  |  |  |
| a. Computed using alpha = .05 | | | | | | | | |

| **Descriptive Statistics** | | | | |
| --- | --- | --- | --- | --- |
|  | group | Mean | Std. Deviation | N |
| LevelMidsGasMP | pronated | 30.2672 | 22.44209 | 30 |
|  | healthy | 29.8627 | 23.73680 | 29 |
|  | Total | 30.0684 | 22.88818 | 59 |
| SandMidGasMP | pronated | 32.5041 | 32.13600 | 30 |
|  | healthy | 32.1699 | 27.18020 | 29 |
|  | Total | 32.3399 | 29.54715 | 59 |

| Source | | Type III Sum of Squares | df | Mean Square | F | Sig. | Partial Eta Squared | Noncent. Parameter |
| --- | --- | --- | --- | --- | --- | --- | --- | --- |
| surface | Sphericity Assumed | 3550.266 | 1 | 3550.266 | 9.883 | .003 | .150 | 9.883 |
|  | Greenhouse-Geisser | 3550.266 | 1.000 | 3550.266 | 9.883 | .003 | .150 | 9.883 |
|  | Huynh-Feldt | 3550.266 | 1.000 | 3550.266 | 9.883 | .003 | .150 | 9.883 |
|  | Lower-bound | 3550.266 | 1.000 | 3550.266 | 9.883 | .003 | .150 | 9.883 |
| surface * group | Sphericity Assumed | 782.106 | 1 | 782.106 | 2.177 | .146 | .037 | 2.177 |
|  | Greenhouse-Geisser | 782.106 | 1.000 | 782.106 | 2.177 | .146 | .037 | 2.177 |
|  | Huynh-Feldt | 782.106 | 1.000 | 782.106 | 2.177 | .146 | .037 | 2.177 |
|  | Lower-bound | 782.106 | 1.000 | 782.106 | 2.177 | .146 | .037 | 2.177 |
| Error(surface) | Sphericity Assumed | 20117.690 | 56 | 359.244 |  |  |  |  |
|  | Greenhouse-Geisser | 20117.690 | 56.000 | 359.244 |  |  |  |  |
|  | Huynh-Feldt | 20117.690 | 56.000 | 359.244 |  |  |  |  |
|  | Lower-bound | 20117.690 | 56.000 | 359.244 |  |  |  |  |

| **Tests of Between-Subjects Effects** | | | | | | | | |
| --- | --- | --- | --- | --- | --- | --- | --- | --- |
| Measure: MEASURE_1 | | | | | | | | |
| Transformed Variable: Average | | | | | | | | |
| Source | Type III Sum of Squares | df | Mean Square | F | Sig. | Partial Eta Squared | Noncent. Parameter | Observed Power^a^ |
| Intercept | 307.515 | 1 | 307.515 | .418 | .520 | .007 | .418 | .097 |
| group | 2961.610 | 1 | 2961.610 | 4.028 | .050 | .067 | 4.028 | .505 |
| Error | 41173.816 | 56 | 735.247 |  |  |  |  |  |
| a. Computed using alpha = .05 | | | | | | | | |

| **Descriptive Statistics** | | | | |
| --- | --- | --- | --- | --- |
|  | group | Mean | Std. Deviation | N |
| LevelMidsGluMP | pronated | 15.7615 | 15.12892 | 30 |
|  | healthy | 19.3229 | 16.83739 | 29 |
|  | Total | 17.5120 | 15.95392 | 59 |
| SandMidGluMP | pronated | 15.1117 | 10.96286 | 30 |
|  | healthy | 21.5366 | 20.19497 | 29 |
|  | Total | 18.2697 | 16.35462 | 59 |

| **Tests of Within-Subjects Effects** | | | | | | | | | |
| --- | --- | --- | --- | --- | --- | --- | --- | --- | --- |
| Measure: MEASURE_1 | | | | | | | | | |
| Source | | Type III Sum of Squares | df | Mean Square | F | Sig. | Partial Eta Squared | Noncent. Parameter | Observed Power^a^ |
| surface | Sphericity Assumed | 175.790 | 1 | 175.790 | 1.614 | .209 | .028 | 1.614 | .239 |
|  | Greenhouse-Geisser | 175.790 | 1.000 | 175.790 | 1.614 | .209 | .028 | 1.614 | .239 |
|  | Huynh-Feldt | 175.790 | 1.000 | 175.790 | 1.614 | .209 | .028 | 1.614 | .239 |
|  | Lower-bound | 175.790 | 1.000 | 175.790 | 1.614 | .209 | .028 | 1.614 | .239 |
| surface * group | Sphericity Assumed | .335 | 1 | .335 | .003 | .956 | .000 | .003 | .050 |
|  | Greenhouse-Geisser | .335 | 1.000 | .335 | .003 | .956 | .000 | .003 | .050 |
|  | Huynh-Feldt | .335 | 1.000 | .335 | .003 | .956 | .000 | .003 | .050 |
|  | Lower-bound | .335 | 1.000 | .335 | .003 | .956 | .000 | .003 | .050 |
| Error(surface) | Sphericity Assumed | 6099.809 | 56 | 108.925 |  |  |  |  |  |
|  | Greenhouse-Geisser | 6099.809 | 56.000 | 108.925 |  |  |  |  |  |
|  | Huynh-Feldt | 6099.809 | 56.000 | 108.925 |  |  |  |  |  |
|  | Lower-bound | 6099.809 | 56.000 | 108.925 |  |  |  |  |  |
| a. Computed using alpha = .05 | | | | | | | | | |

| **Tests of Between-Subjects Effects** | | | | | | | | |
| --- | --- | --- | --- | --- | --- | --- | --- | --- |
| Measure: MEASURE_1 | | | | | | | | |
| Transformed Variable: Average | | | | | | | | |
| Source | Type III Sum of Squares | df | Mean Square | F | Sig. | Partial Eta Squared | Noncent. Parameter | Observed Power^a^ |
| Intercept | 1864.751 | 1 | 1864.751 | 4.558 | .037 | .075 | 4.558 | .555 |
| group | 324.267 | 1 | 324.267 | .793 | .377 | .014 | .793 | .141 |
| Error | 22912.200 | 56 | 409.146 |  |  |  |  |  |
| a. Computed using alpha = .05 | | | | | | | | |

| **Descriptive Statistics** | | | | |
| --- | --- | --- | --- | --- |
|  | group | Mean | Std. Deviation | N |
| LevelMidsRFP | pronated | 16.8289 | 9.30612 | 30 |
|  | healthy | 20.4537 | 15.35882 | 29 |
|  | Total | 18.6106 | 12.66972 | 59 |
| SandMidRFP | pronated | 18.7130 | 11.66331 | 30 |
|  | healthy | 20.7305 | 15.66510 | 29 |
|  | Total | 19.7047 | 13.69372 | 59 |

| **Tests of Within-Subjects Effects** | | | | | | | | | |
| --- | --- | --- | --- | --- | --- | --- | --- | --- | --- |
| Measure: MEASURE_1 | | | | | | | | | |
| Source | | Type III Sum of Squares | df | Mean Square | F | Sig. | Partial Eta Squared | Noncent. Parameter | Observed Power^a^ |
| surface | Sphericity Assumed | 19.594 | 1 | 19.594 | .473 | .495 | .008 | .473 | .104 |
|  | Greenhouse-Geisser | 19.594 | 1.000 | 19.594 | .473 | .495 | .008 | .473 | .104 |
|  | Huynh-Feldt | 19.594 | 1.000 | 19.594 | .473 | .495 | .008 | .473 | .104 |
|  | Lower-bound | 19.594 | 1.000 | 19.594 | .473 | .495 | .008 | .473 | .104 |
| surface * group | Sphericity Assumed | 7.604 | 1 | 7.604 | .183 | .670 | .003 | .183 | .071 |
|  | Greenhouse-Geisser | 7.604 | 1.000 | 7.604 | .183 | .670 | .003 | .183 | .071 |
|  | Huynh-Feldt | 7.604 | 1.000 | 7.604 | .183 | .670 | .003 | .183 | .071 |
|  | Lower-bound | 7.604 | 1.000 | 7.604 | .183 | .670 | .003 | .183 | .071 |
| Error(surface) | Sphericity Assumed | 2320.815 | 56 | 41.443 |  |  |  |  |  |
|  | Greenhouse-Geisser | 2320.815 | 56.000 | 41.443 |  |  |  |  |  |
|  | Huynh-Feldt | 2320.815 | 56.000 | 41.443 |  |  |  |  |  |
|  | Lower-bound | 2320.815 | 56.000 | 41.443 |  |  |  |  |  |
| a. Computed using alpha = .05 | | | | | | | | | |

| **Tests of Between-Subjects Effects** | | | | | | | | |
| --- | --- | --- | --- | --- | --- | --- | --- | --- |
| Measure: MEASURE_1 | | | | | | | | |
| Transformed Variable: Average | | | | | | | | |
| Source | Type III Sum of Squares | df | Mean Square | F | Sig. | Partial Eta Squared | Noncent. Parameter | Observed Power^a^ |
| Intercept | 499.152 | 1 | 499.152 | 1.760 | .190 | .030 | 1.760 | .256 |
| group | 13.952 | 1 | 13.952 | .049 | .825 | .001 | .049 | .055 |
| Error | 15886.203 | 56 | 283.682 |  |  |  |  |  |
| a. Computed using alpha = .05 | | | | | | | | |

| **Descriptive Statistics** | | | | |
| --- | --- | --- | --- | --- |
|  | group | Mean | Std. Deviation | N |
| LevelMidsSTP | pronated | 12.3289 | 19.06015 | 30 |
|  | healthy | 7.3868 | 6.21872 | 29 |
|  | Total | 9.8998 | 14.37094 | 59 |
| SandMidSTP | pronated | 9.9700 | 11.11778 | 30 |
|  | healthy | 7.1260 | 6.27360 | 29 |
|  | Total | 8.5721 | 9.10271 | 59 |

| **Tests of Within-Subjects Effects** | | | | | | | | | |
| --- | --- | --- | --- | --- | --- | --- | --- | --- | --- |
| Measure: MEASURE_1 | | | | | | | | | |
| Source | | Type III Sum of Squares | df | Mean Square | F | Sig. | Partial Eta Squared | Noncent. Parameter | Observed Power^a^ |
| surface | Sphericity Assumed | 5.900 | 1 | 5.900 | .056 | .814 | .001 | .056 | .056 |
|  | Greenhouse-Geisser | 5.900 | 1.000 | 5.900 | .056 | .814 | .001 | .056 | .056 |
|  | Huynh-Feldt | 5.900 | 1.000 | 5.900 | .056 | .814 | .001 | .056 | .056 |
|  | Lower-bound | 5.900 | 1.000 | 5.900 | .056 | .814 | .001 | .056 | .056 |
| surface * group | Sphericity Assumed | 51.532 | 1 | 51.532 | .487 | .488 | .009 | .487 | .105 |
|  | Greenhouse-Geisser | 51.532 | 1.000 | 51.532 | .487 | .488 | .009 | .487 | .105 |
|  | Huynh-Feldt | 51.532 | 1.000 | 51.532 | .487 | .488 | .009 | .487 | .105 |
|  | Lower-bound | 51.532 | 1.000 | 51.532 | .487 | .488 | .009 | .487 | .105 |
| Error(surface) | Sphericity Assumed | 5924.763 | 56 | 105.799 |  |  |  |  |  |
|  | Greenhouse-Geisser | 5924.763 | 56.000 | 105.799 |  |  |  |  |  |
|  | Huynh-Feldt | 5924.763 | 56.000 | 105.799 |  |  |  |  |  |
|  | Lower-bound | 5924.763 | 56.000 | 105.799 |  |  |  |  |  |
| a. Computed using alpha = .05 | | | | | | | | | |

| **Tests of Between-Subjects Effects** | | | | | | | | |
| --- | --- | --- | --- | --- | --- | --- | --- | --- |
| Measure: MEASURE_1 | | | | | | | | |
| Transformed Variable: Average | | | | | | | | |
| Source | Type III Sum of Squares | df | Mean Square | F | Sig. | Partial Eta Squared | Noncent. Parameter | Observed Power^a^ |
| Intercept | 1162.787 | 1 | 1162.787 | 6.300 | .015 | .101 | 6.300 | .694 |
| group | 295.305 | 1 | 295.305 | 1.600 | .211 | .028 | 1.600 | .237 |
| Error | 10336.208 | 56 | 184.575 |  |  |  |  |  |
| a. Computed using alpha = .05 | | | | | | | | |

| **Descriptive Statistics** | | | | |
| --- | --- | --- | --- | --- |
|  | group | Mean | Std. Deviation | N |
| LevelMidsTAP | pronated | 8.6440 | 7.28004 | 30 |
|  | healthy | 10.7408 | 11.31366 | 29 |
|  | Total | 9.6746 | 9.45567 | 59 |
| SandMidTAP | pronated | 10.3221 | 11.16515 | 30 |
|  | healthy | 12.8547 | 11.95073 | 29 |
|  | Total | 11.5670 | 11.52860 | 59 |

| **Tests of Within-Subjects Effects** | | | | | | | | | |
| --- | --- | --- | --- | --- | --- | --- | --- | --- | --- |
| Measure: MEASURE_1 | | | | | | | | | |
| Source | | Type III Sum of Squares | df | Mean Square | F | Sig. | Partial Eta Squared | Noncent. Parameter | Observed Power^a^ |
| surface | Sphericity Assumed | 9.501 | 1 | 9.501 | .213 | .646 | .004 | .213 | .074 |
|  | Greenhouse-Geisser | 9.501 | 1.000 | 9.501 | .213 | .646 | .004 | .213 | .074 |
|  | Huynh-Feldt | 9.501 | 1.000 | 9.501 | .213 | .646 | .004 | .213 | .074 |
|  | Lower-bound | 9.501 | 1.000 | 9.501 | .213 | .646 | .004 | .213 | .074 |
| surface * group | Sphericity Assumed | 2.759 | 1 | 2.759 | .062 | .804 | .001 | .062 | .057 |
|  | Greenhouse-Geisser | 2.759 | 1.000 | 2.759 | .062 | .804 | .001 | .062 | .057 |
|  | Huynh-Feldt | 2.759 | 1.000 | 2.759 | .062 | .804 | .001 | .062 | .057 |
|  | Lower-bound | 2.759 | 1.000 | 2.759 | .062 | .804 | .001 | .062 | .057 |
| Error(surface) | Sphericity Assumed | 2495.725 | 56 | 44.567 |  |  |  |  |  |
|  | Greenhouse-Geisser | 2495.725 | 56.000 | 44.567 |  |  |  |  |  |
|  | Huynh-Feldt | 2495.725 | 56.000 | 44.567 |  |  |  |  |  |
|  | Lower-bound | 2495.725 | 56.000 | 44.567 |  |  |  |  |  |
| a. Computed using alpha = .05 | | | | | | | | | |

| **Tests of Between-Subjects Effects** | | | | | | | | |
| --- | --- | --- | --- | --- | --- | --- | --- | --- |
| Measure: MEASURE_1 | | | | | | | | |
| Transformed Variable: Average | | | | | | | | |
| Source | Type III Sum of Squares | df | Mean Square | F | Sig. | Partial Eta Squared | Noncent. Parameter | Observed Power^a^ |
| Intercept | 425.745 | 1 | 425.745 | 3.464 | .068 | .058 | 3.464 | .448 |
| group | 171.060 | 1 | 171.060 | 1.392 | .243 | .024 | 1.392 | .213 |
| Error | 6882.510 | 56 | 122.902 |  |  |  |  |  |
| a. Computed using alpha = .05 | | | | | | | | |

| **Descriptive Statistics** | | | | |
| --- | --- | --- | --- | --- |
|  | group | Mean | Std. Deviation | N |
| LevelMidsVLP | pronated | 11.9231 | 13.19630 | 30 |
|  | healthy | 7.5788 | 6.02893 | 29 |
|  | Total | 9.7878 | 10.46024 | 59 |
| SandMidVLP | pronated | 14.0358 | 17.83890 | 30 |
|  | healthy | 13.1554 | 19.24644 | 29 |
|  | Total | 13.6031 | 18.38849 | 59 |

| Source | | Type III Sum of Squares | df | Mean Square | F | Sig. | Partial Eta Squared | Noncent. Parameter |
| --- | --- | --- | --- | --- | --- | --- | --- | --- |
| surface | Sphericity Assumed | 348.145 | 1 | 348.145 | 2.434 | .124 | .042 | 2.434 |
|  | Greenhouse-Geisser | 348.145 | 1.000 | 348.145 | 2.434 | .124 | .042 | 2.434 |
|  | Huynh-Feldt | 348.145 | 1.000 | 348.145 | 2.434 | .124 | .042 | 2.434 |
|  | Lower-bound | 348.145 | 1.000 | 348.145 | 2.434 | .124 | .042 | 2.434 |
| surface * group | Sphericity Assumed | 6.486 | 1 | 6.486 | .045 | .832 | .001 | .045 |
|  | Greenhouse-Geisser | 6.486 | 1.000 | 6.486 | .045 | .832 | .001 | .045 |
|  | Huynh-Feldt | 6.486 | 1.000 | 6.486 | .045 | .832 | .001 | .045 |
|  | Lower-bound | 6.486 | 1.000 | 6.486 | .045 | .832 | .001 | .045 |
| Error(surface) | Sphericity Assumed | 8009.112 | 56 | 143.020 |  |  |  |  |
|  | Greenhouse-Geisser | 8009.112 | 56.000 | 143.020 |  |  |  |  |
|  | Huynh-Feldt | 8009.112 | 56.000 | 143.020 |  |  |  |  |
|  | Lower-bound | 8009.112 | 56.000 | 143.020 |  |  |  |  |

| **Tests of Between-Subjects Effects** | | | | | | | | |
| --- | --- | --- | --- | --- | --- | --- | --- | --- |
| Measure: MEASURE_1 | | | | | | | | |
| Transformed Variable: Average | | | | | | | | |
| Source | Type III Sum of Squares | df | Mean Square | F | Sig. | Partial Eta Squared | Noncent. Parameter | Observed Power^a^ |
| Intercept | 45.202 | 1 | 45.202 | .159 | .692 | .003 | .159 | .068 |
| group | 708.342 | 1 | 708.342 | 2.490 | .120 | .043 | 2.490 | .341 |
| Error | 15930.093 | 56 | 284.466 |  |  |  |  |  |
| a. Computed using alpha = .05 | | | | | | | | |

| **Descriptive Statistics** | | | | |
| --- | --- | --- | --- | --- |
|  | group | Mean | Std. Deviation | N |
| LevelMidsVMP | pronated | 12.2214 | 12.64812 | 30 |
|  | healthy | 18.8667 | 22.63876 | 29 |
|  | Total | 15.4878 | 18.40204 | 59 |
| SandMidVMP | pronated | 11.8808 | 13.13921 | 30 |
|  | healthy | 21.4333 | 17.05397 | 29 |
|  | Total | 16.5761 | 15.80895 | 59 |

| Source | | Type III Sum of Squares | df | Mean Square | F | Sig. | Partial Eta Squared | Noncent. Parameter |
| --- | --- | --- | --- | --- | --- | --- | --- | --- |
| surface | Sphericity Assumed | 265.150 | 1 | 265.150 | 1.206 | .277 | .021 | 1.206 |
|  | Greenhouse-Geisser | 265.150 | 1.000 | 265.150 | 1.206 | .277 | .021 | 1.206 |
|  | Huynh-Feldt | 265.150 | 1.000 | 265.150 | 1.206 | .277 | .021 | 1.206 |
|  | Lower-bound | 265.150 | 1.000 | 265.150 | 1.206 | .277 | .021 | 1.206 |
| surface * group | Sphericity Assumed | 184.723 | 1 | 184.723 | .840 | .363 | .015 | .840 |
|  | Greenhouse-Geisser | 184.723 | 1.000 | 184.723 | .840 | .363 | .015 | .840 |
|  | Huynh-Feldt | 184.723 | 1.000 | 184.723 | .840 | .363 | .015 | .840 |
|  | Lower-bound | 184.723 | 1.000 | 184.723 | .840 | .363 | .015 | .840 |
| Error(surface) | Sphericity Assumed | 12312.730 | 56 | 219.870 |  |  |  |  |
|  | Greenhouse-Geisser | 12312.730 | 56.000 | 219.870 |  |  |  |  |
|  | Huynh-Feldt | 12312.730 | 56.000 | 219.870 |  |  |  |  |
|  | Lower-bound | 12312.730 | 56.000 | 219.870 |  |  |  |  |

| **Tests of Between-Subjects Effects** | | | | | | | | |
| --- | --- | --- | --- | --- | --- | --- | --- | --- |
| Measure: MEASURE_1 | | | | | | | | |
| Transformed Variable: Average | | | | | | | | |
| Source | Type III Sum of Squares | df | Mean Square | F | Sig. | Partial Eta Squared | Noncent. Parameter | Observed Power^a^ |
| Intercept | 502.274 | 1 | 502.274 | 1.509 | .224 | .026 | 1.509 | .227 |
| group | 711.144 | 1 | 711.144 | 2.137 | .149 | .037 | 2.137 | .301 |
| Error | 18638.422 | 56 | 332.829 |  |  |  |  |  |
| a. Computed using alpha = .05 | | | | | | | | |

| **Descriptive Statistics** | | | | |
| --- | --- | --- | --- | --- |
|  | group | Mean | Std. Deviation | N |
| LevelPOBFP | pronated | 5.8337 | 8.83767 | 30 |
|  | healthy | 7.3557 | 7.37462 | 29 |
|  | Total | 6.5818 | 8.11763 | 59 |
| SandPOBFP | pronated | 4.2845 | 3.07433 | 30 |
|  | healthy | 7.9666 | 9.61418 | 29 |
|  | Total | 6.0944 | 7.26603 | 59 |

| Source | | Type III Sum of Squares | df | Mean Square | F | Sig. | Partial Eta Squared | Noncent. Parameter |
| --- | --- | --- | --- | --- | --- | --- | --- | --- |
| surface | Sphericity Assumed | .690 | 1 | .690 | .016 | .900 | .000 | .016 |
|  | Greenhouse-Geisser | .690 | 1.000 | .690 | .016 | .900 | .000 | .016 |
|  | Huynh-Feldt | .690 | 1.000 | .690 | .016 | .900 | .000 | .016 |
|  | Lower-bound | .690 | 1.000 | .690 | .016 | .900 | .000 | .016 |
| surface * group | Sphericity Assumed | 36.193 | 1 | 36.193 | .833 | .365 | .015 | .833 |
|  | Greenhouse-Geisser | 36.193 | 1.000 | 36.193 | .833 | .365 | .015 | .833 |
|  | Huynh-Feldt | 36.193 | 1.000 | 36.193 | .833 | .365 | .015 | .833 |
|  | Lower-bound | 36.193 | 1.000 | 36.193 | .833 | .365 | .015 | .833 |
| Error(surface) | Sphericity Assumed | 2434.310 | 56 | 43.470 |  |  |  |  |
|  | Greenhouse-Geisser | 2434.310 | 56.000 | 43.470 |  |  |  |  |
|  | Huynh-Feldt | 2434.310 | 56.000 | 43.470 |  |  |  |  |
|  | Lower-bound | 2434.310 | 56.000 | 43.470 |  |  |  |  |

| **Tests of Between-Subjects Effects** | | | | | | | | |
| --- | --- | --- | --- | --- | --- | --- | --- | --- |
| Measure: MEASURE_1 | | | | | | | | |
| Transformed Variable: Average | | | | | | | | |
| Source | Type III Sum of Squares | df | Mean Square | F | Sig. | Partial Eta Squared | Noncent. Parameter | Observed Power^a^ |
| Intercept | 6.593 | 1 | 6.593 | .102 | .751 | .002 | .102 | .061 |
| group | 6.337 | 1 | 6.337 | .098 | .756 | .002 | .098 | .061 |
| Error | 3627.803 | 56 | 64.782 |  |  |  |  |  |
| a. Computed using alpha = .05 | | | | | | | | |

| **Descriptive Statistics** | | | | |
| --- | --- | --- | --- | --- |
|  | group | Mean | Std. Deviation | N |
| LevelPOGasMP | pronated | 46.9519 | 23.74832 | 30 |
|  | healthy | 37.7444 | 22.44029 | 29 |
|  | Total | 42.4262 | 23.38047 | 59 |
| SandPOGasMP | pronated | 51.0733 | 23.05600 | 30 |
|  | healthy | 36.2321 | 19.87846 | 29 |
|  | Total | 43.7785 | 22.63960 | 59 |

| Source | | Type III Sum of Squares | df | Mean Square | F | Sig. | Partial Eta Squared | Noncent. Parameter |
| --- | --- | --- | --- | --- | --- | --- | --- | --- |
| surface | Sphericity Assumed | 272.520 | 1 | 272.520 | 1.010 | .319 | .018 | 1.010 |
|  | Greenhouse-Geisser | 272.520 | 1.000 | 272.520 | 1.010 | .319 | .018 | 1.010 |
|  | Huynh-Feldt | 272.520 | 1.000 | 272.520 | 1.010 | .319 | .018 | 1.010 |
|  | Lower-bound | 272.520 | 1.000 | 272.520 | 1.010 | .319 | .018 | 1.010 |
| surface * group | Sphericity Assumed | 55.367 | 1 | 55.367 | .205 | .652 | .004 | .205 |
|  | Greenhouse-Geisser | 55.367 | 1.000 | 55.367 | .205 | .652 | .004 | .205 |
|  | Huynh-Feldt | 55.367 | 1.000 | 55.367 | .205 | .652 | .004 | .205 |
|  | Lower-bound | 55.367 | 1.000 | 55.367 | .205 | .652 | .004 | .205 |
| Error(surface) | Sphericity Assumed | 15108.198 | 56 | 269.789 |  |  |  |  |
|  | Greenhouse-Geisser | 15108.198 | 56.000 | 269.789 |  |  |  |  |
|  | Huynh-Feldt | 15108.198 | 56.000 | 269.789 |  |  |  |  |
|  | Lower-bound | 15108.198 | 56.000 | 269.789 |  |  |  |  |

| **Tests of Between-Subjects Effects** | | | | | | | | |
| --- | --- | --- | --- | --- | --- | --- | --- | --- |
| Measure: MEASURE_1 | | | | | | | | |
| Transformed Variable: Average | | | | | | | | |
| Source | Type III Sum of Squares | df | Mean Square | F | Sig. | Partial Eta Squared | Noncent. Parameter | Observed Power^a^ |
| Intercept | 47676.963 | 1 | 47676.963 | 77.037 | .000 | .579 | 77.037 | 1.000 |
| group | 562.792 | 1 | 562.792 | .909 | .344 | .016 | .909 | .155 |
| Error | 34657.576 | 56 | 618.885 |  |  |  |  |  |
| a. Computed using alpha = .05 | | | | | | | | |

| **Descriptive Statistics** | | | | |
| --- | --- | --- | --- | --- |
|  | group | Mean | Std. Deviation | N |
| LevelPOGluMP | pronated | 12.1397 | 8.71959 | 30 |
|  | healthy | 16.8586 | 17.94909 | 29 |
|  | Total | 14.4591 | 14.11407 | 59 |
| SandPOGluMP | pronated | 8.3043 | 6.22286 | 30 |
|  | healthy | 19.8871 | 14.97141 | 29 |
|  | Total | 13.9975 | 12.71528 | 59 |

| Source | | Type III Sum of Squares | df | Mean Square | F | Sig. | Partial Eta Squared | Noncent. Parameter |
| --- | --- | --- | --- | --- | --- | --- | --- | --- |
| surface | Sphericity Assumed | 57.975 | 1 | 57.975 | .406 | .527 | .007 | .406 |
|  | Greenhouse-Geisser | 57.975 | 1.000 | 57.975 | .406 | .527 | .007 | .406 |
|  | Huynh-Feldt | 57.975 | 1.000 | 57.975 | .406 | .527 | .007 | .406 |
|  | Lower-bound | 57.975 | 1.000 | 57.975 | .406 | .527 | .007 | .406 |
| surface * group | Sphericity Assumed | 189.756 | 1 | 189.756 | 1.329 | .254 | .023 | 1.329 |
|  | Greenhouse-Geisser | 189.756 | 1.000 | 189.756 | 1.329 | .254 | .023 | 1.329 |
|  | Huynh-Feldt | 189.756 | 1.000 | 189.756 | 1.329 | .254 | .023 | 1.329 |
|  | Lower-bound | 189.756 | 1.000 | 189.756 | 1.329 | .254 | .023 | 1.329 |
| Error(surface) | Sphericity Assumed | 7995.328 | 56 | 142.774 |  |  |  |  |
|  | Greenhouse-Geisser | 7995.328 | 56.000 | 142.774 |  |  |  |  |
|  | Huynh-Feldt | 7995.328 | 56.000 | 142.774 |  |  |  |  |
|  | Lower-bound | 7995.328 | 56.000 | 142.774 |  |  |  |  |

| **Tests of Between-Subjects Effects** | | | | | | | | |
| --- | --- | --- | --- | --- | --- | --- | --- | --- |
| Measure: MEASURE_1 | | | | | | | | |
| Transformed Variable: Average | | | | | | | | |
| Source | Type III Sum of Squares | df | Mean Square | F | Sig. | Partial Eta Squared | Noncent. Parameter | Observed Power^a^ |
| Intercept | 33.835 | 1 | 33.835 | .249 | .620 | .004 | .249 | .078 |
| group | 287.434 | 1 | 287.434 | 2.114 | .152 | .036 | 2.114 | .298 |
| Error | 7614.138 | 56 | 135.967 |  |  |  |  |  |
| a. Computed using alpha = .05 | | | | | | | | |

| **Descriptive Statistics** | | | | |
| --- | --- | --- | --- | --- |
|  | group | Mean | Std. Deviation | N |
| LevelPORFP | pronated | 17.8316 | 11.98646 | 30 |
|  | healthy | 20.5905 | 14.34852 | 29 |
|  | Total | 19.1877 | 13.15914 | 59 |
| SandPORFP | pronated | 15.6131 | 7.49067 | 30 |
|  | healthy | 21.2442 | 15.73296 | 29 |
|  | Total | 18.3809 | 12.47446 | 59 |

| Source | | Type III Sum of Squares | df | Mean Square | F | Sig. | Partial Eta Squared | Noncent. Parameter |
| --- | --- | --- | --- | --- | --- | --- | --- | --- |
| surface | Sphericity Assumed | 3.292 | 1 | 3.292 | .124 | .726 | .002 | .124 |
|  | Greenhouse-Geisser | 3.292 | 1.000 | 3.292 | .124 | .726 | .002 | .124 |
|  | Huynh-Feldt | 3.292 | 1.000 | 3.292 | .124 | .726 | .002 | .124 |
|  | Lower-bound | 3.292 | 1.000 | 3.292 | .124 | .726 | .002 | .124 |
| surface * group | Sphericity Assumed | 46.446 | 1 | 46.446 | 1.751 | .191 | .030 | 1.751 |
|  | Greenhouse-Geisser | 46.446 | 1.000 | 46.446 | 1.751 | .191 | .030 | 1.751 |
|  | Huynh-Feldt | 46.446 | 1.000 | 46.446 | 1.751 | .191 | .030 | 1.751 |
|  | Lower-bound | 46.446 | 1.000 | 46.446 | 1.751 | .191 | .030 | 1.751 |
| Error(surface) | Sphericity Assumed | 1485.463 | 56 | 26.526 |  |  |  |  |
|  | Greenhouse-Geisser | 1485.463 | 56.000 | 26.526 |  |  |  |  |
|  | Huynh-Feldt | 1485.463 | 56.000 | 26.526 |  |  |  |  |
|  | Lower-bound | 1485.463 | 56.000 | 26.526 |  |  |  |  |

| **Tests of Between-Subjects Effects** | | | | | | | | |
| --- | --- | --- | --- | --- | --- | --- | --- | --- |
| Measure: MEASURE_1 | | | | | | | | |
| Transformed Variable: Average | | | | | | | | |
| Source | Type III Sum of Squares | df | Mean Square | F | Sig. | Partial Eta Squared | Noncent. Parameter | Observed Power^a^ |
| Intercept | 161.198 | 1 | 161.198 | .624 | .433 | .011 | .624 | .121 |
| group | .566 | 1 | .566 | .002 | .963 | .000 | .002 | .050 |
| Error | 14470.922 | 56 | 258.409 |  |  |  |  |  |
| a. Computed using alpha = .05 | | | | | | | | |

| **Descriptive Statistics** | | | | |
| --- | --- | --- | --- | --- |
|  | group | Mean | Std. Deviation | N |
| LevelPOSTP | pronated | 7.8720 | 11.74204 | 30 |
|  | healthy | 8.2825 | 14.40387 | 29 |
|  | Total | 8.0738 | 13.00536 | 59 |
| SandPOSTP | pronated | 4.9916 | 5.13795 | 30 |
|  | healthy | 7.3545 | 6.93167 | 29 |
|  | Total | 6.1530 | 6.14934 | 59 |

| Source | | Type III Sum of Squares | df | Mean Square | F | Sig. | Partial Eta Squared | Noncent. Parameter |
| --- | --- | --- | --- | --- | --- | --- | --- | --- |
| surface | Sphericity Assumed | 4.569 | 1 | 4.569 | .047 | .829 | .001 | .047 |
|  | Greenhouse-Geisser | 4.569 | 1.000 | 4.569 | .047 | .829 | .001 | .047 |
|  | Huynh-Feldt | 4.569 | 1.000 | 4.569 | .047 | .829 | .001 | .047 |
|  | Lower-bound | 4.569 | 1.000 | 4.569 | .047 | .829 | .001 | .047 |
| surface * group | Sphericity Assumed | 27.206 | 1 | 27.206 | .281 | .598 | .005 | .281 |
|  | Greenhouse-Geisser | 27.206 | 1.000 | 27.206 | .281 | .598 | .005 | .281 |
|  | Huynh-Feldt | 27.206 | 1.000 | 27.206 | .281 | .598 | .005 | .281 |
|  | Lower-bound | 27.206 | 1.000 | 27.206 | .281 | .598 | .005 | .281 |
| Error(surface) | Sphericity Assumed | 5417.202 | 56 | 96.736 |  |  |  |  |
|  | Greenhouse-Geisser | 5417.202 | 56.000 | 96.736 |  |  |  |  |
|  | Huynh-Feldt | 5417.202 | 56.000 | 96.736 |  |  |  |  |
|  | Lower-bound | 5417.202 | 56.000 | 96.736 |  |  |  |  |

| **Tests of Between-Subjects Effects** | | | | | | | | |
| --- | --- | --- | --- | --- | --- | --- | --- | --- |
| Measure: MEASURE_1 | | | | | | | | |
| Transformed Variable: Average | | | | | | | | |
| Source | Type III Sum of Squares | df | Mean Square | F | Sig. | Partial Eta Squared | Noncent. Parameter | Observed Power^a^ |
| Intercept | 17.708 | 1 | 17.708 | .162 | .689 | .003 | .162 | .068 |
| group | 2.378 | 1 | 2.378 | .022 | .883 | .000 | .022 | .052 |
| Error | 6113.059 | 56 | 109.162 |  |  |  |  |  |
| a. Computed using alpha = .05 | | | | | | | | |

| **Descriptive Statistics** | | | | |
| --- | --- | --- | --- | --- |
|  | group | Mean | Std. Deviation | N |
| LevelPOTAP | pronated | 9.0103 | 6.55923 | 30 |
|  | healthy | 11.3394 | 10.19722 | 29 |
|  | Total | 10.1551 | 8.54926 | 59 |
| SandPOTAP | pronated | 8.8679 | 6.07085 | 30 |
|  | healthy | 12.9165 | 13.99807 | 29 |
|  | Total | 10.8579 | 10.82541 | 59 |

| Source | | Type III Sum of Squares | df | Mean Square | F | Sig. | Partial Eta Squared | Noncent. Parameter |
| --- | --- | --- | --- | --- | --- | --- | --- | --- |
| surface | Sphericity Assumed | 55.353 | 1 | 55.353 | 1.614 | .209 | .028 | 1.614 |
|  | Greenhouse-Geisser | 55.353 | 1.000 | 55.353 | 1.614 | .209 | .028 | 1.614 |
|  | Huynh-Feldt | 55.353 | 1.000 | 55.353 | 1.614 | .209 | .028 | 1.614 |
|  | Lower-bound | 55.353 | 1.000 | 55.353 | 1.614 | .209 | .028 | 1.614 |
| surface * group | Sphericity Assumed | .162 | 1 | .162 | .005 | .946 | .000 | .005 |
|  | Greenhouse-Geisser | .162 | 1.000 | .162 | .005 | .946 | .000 | .005 |
|  | Huynh-Feldt | .162 | 1.000 | .162 | .005 | .946 | .000 | .005 |
|  | Lower-bound | .162 | 1.000 | .162 | .005 | .946 | .000 | .005 |
| Error(surface) | Sphericity Assumed | 1920.851 | 56 | 34.301 |  |  |  |  |
|  | Greenhouse-Geisser | 1920.851 | 56.000 | 34.301 |  |  |  |  |
|  | Huynh-Feldt | 1920.851 | 56.000 | 34.301 |  |  |  |  |
|  | Lower-bound | 1920.851 | 56.000 | 34.301 |  |  |  |  |

| **Tests of Between-Subjects Effects** | | | | | | | | |
| --- | --- | --- | --- | --- | --- | --- | --- | --- |
| Measure: MEASURE_1 | | | | | | | | |
| Transformed Variable: Average | | | | | | | | |
| Source | Type III Sum of Squares | df | Mean Square | F | Sig. | Partial Eta Squared | Noncent. Parameter | Observed Power^a^ |
| Intercept | 146.596 | 1 | 146.596 | 1.287 | .262 | .022 | 1.287 | .200 |
| group | 23.375 | 1 | 23.375 | .205 | .652 | .004 | .205 | .073 |
| Error | 6380.728 | 56 | 113.942 |  |  |  |  |  |
| a. Computed using alpha = .05 | | | | | | | | |

| **Descriptive Statistics** | | | | |
| --- | --- | --- | --- | --- |
|  | group | Mean | Std. Deviation | N |
| LevelPOVLP | pronated | 8.8504 | 12.56334 | 30 |
|  | healthy | 7.1733 | 9.15834 | 29 |
|  | Total | 8.0261 | 10.96017 | 59 |
| SandPOVLP | pronated | 10.1997 | 15.87057 | 30 |
|  | healthy | 10.0448 | 12.32517 | 29 |
|  | Total | 10.1236 | 14.11663 | 59 |

| Source | | Type III Sum of Squares | df | Mean Square | F | Sig. | Partial Eta Squared | Noncent. Parameter |
| --- | --- | --- | --- | --- | --- | --- | --- | --- |
| surface | Sphericity Assumed | 135.790 | 1 | 135.790 | 2.426 | .125 | .042 | 2.426 |
|  | Greenhouse-Geisser | 135.790 | 1.000 | 135.790 | 2.426 | .125 | .042 | 2.426 |
|  | Huynh-Feldt | 135.790 | 1.000 | 135.790 | 2.426 | .125 | .042 | 2.426 |
|  | Lower-bound | 135.790 | 1.000 | 135.790 | 2.426 | .125 | .042 | 2.426 |
| surface * group | Sphericity Assumed | 8.747 | 1 | 8.747 | .156 | .694 | .003 | .156 |
|  | Greenhouse-Geisser | 8.747 | 1.000 | 8.747 | .156 | .694 | .003 | .156 |
|  | Huynh-Feldt | 8.747 | 1.000 | 8.747 | .156 | .694 | .003 | .156 |
|  | Lower-bound | 8.747 | 1.000 | 8.747 | .156 | .694 | .003 | .156 |
| Error(surface) | Sphericity Assumed | 3134.992 | 56 | 55.982 |  |  |  |  |
|  | Greenhouse-Geisser | 3134.992 | 56.000 | 55.982 |  |  |  |  |
|  | Huynh-Feldt | 3134.992 | 56.000 | 55.982 |  |  |  |  |
|  | Lower-bound | 3134.992 | 56.000 | 55.982 |  |  |  |  |

| **Tests of Between-Subjects Effects** | | | | | | | | |
| --- | --- | --- | --- | --- | --- | --- | --- | --- |
| Measure: MEASURE_1 | | | | | | | | |
| Transformed Variable: Average | | | | | | | | |
| Source | Type III Sum of Squares | df | Mean Square | F | Sig. | Partial Eta Squared | Noncent. Parameter | Observed Power^a^ |
| Intercept | 628.233 | 1 | 628.233 | 2.966 | .091 | .050 | 2.966 | .395 |
| group | 822.191 | 1 | 822.191 | 3.881 | .054 | .065 | 3.881 | .491 |
| Error | 11862.101 | 56 | 211.823 |  |  |  |  |  |
| a. Computed using alpha = .05 | | | | | | | | |

| **Descriptive Statistics** | | | | |
| --- | --- | --- | --- | --- |
|  | group | Mean | Std. Deviation | N |
| LevelPOVMP | pronated | 14.6470 | 16.20538 | 30 |
|  | healthy | 18.3920 | 19.19187 | 29 |
|  | Total | 16.4877 | 17.68294 | 59 |
| SandPOVMP | pronated | 17.4099 | 22.01976 | 30 |
|  | healthy | 18.0795 | 16.01604 | 29 |
|  | Total | 17.7390 | 19.14113 | 59 |

| Source | | Type III Sum of Squares | df | Mean Square | F | Sig. | Partial Eta Squared | Noncent. Parameter |
| --- | --- | --- | --- | --- | --- | --- | --- | --- |
| surface | Sphericity Assumed | 230.326 | 1 | 230.326 | 1.015 | .318 | .018 | 1.015 |
|  | Greenhouse-Geisser | 230.326 | 1.000 | 230.326 | 1.015 | .318 | .018 | 1.015 |
|  | Huynh-Feldt | 230.326 | 1.000 | 230.326 | 1.015 | .318 | .018 | 1.015 |
|  | Lower-bound | 230.326 | 1.000 | 230.326 | 1.015 | .318 | .018 | 1.015 |
| surface * group | Sphericity Assumed | 2.849 | 1 | 2.849 | .013 | .911 | .000 | .013 |
|  | Greenhouse-Geisser | 2.849 | 1.000 | 2.849 | .013 | .911 | .000 | .013 |
|  | Huynh-Feldt | 2.849 | 1.000 | 2.849 | .013 | .911 | .000 | .013 |
|  | Lower-bound | 2.849 | 1.000 | 2.849 | .013 | .911 | .000 | .013 |
| Error(surface) | Sphericity Assumed | 12711.585 | 56 | 226.993 |  |  |  |  |
|  | Greenhouse-Geisser | 12711.585 | 56.000 | 226.993 |  |  |  |  |
|  | Huynh-Feldt | 12711.585 | 56.000 | 226.993 |  |  |  |  |
|  | Lower-bound | 12711.585 | 56.000 | 226.993 |  |  |  |  |

| **Tests of Between-Subjects Effects** | | | | | | | | |
| --- | --- | --- | --- | --- | --- | --- | --- | --- |
| Measure: MEASURE_1 | | | | | | | | |
| Transformed Variable: Average | | | | | | | | |
| Source | Type III Sum of Squares | df | Mean Square | F | Sig. | Partial Eta Squared | Noncent. Parameter | Observed Power^a^ |
| Intercept | 33.128 | 1 | 33.128 | .084 | .773 | .001 | .084 | .059 |
| group | 266.010 | 1 | 266.010 | .672 | .416 | .012 | .672 | .127 |
| Error | 22181.221 | 56 | 396.093 |  |  |  |  |  |
| a. Computed using alpha = .05 | | | | | | | | |

| **Descriptive Statistics** | | | | |
| --- | --- | --- | --- | --- |
|  | group | Mean | Std. Deviation | N |
| LevelsiwnBFP | pronated | 7.6545 | 9.96370 | 30 |
|  | healthy | 8.4121 | 6.20168 | 29 |
|  | Total | 8.0269 | 8.26746 | 59 |
| SandsiwnBFP | pronated | 6.0748 | 4.32756 | 30 |
|  | healthy | 7.7618 | 5.49905 | 29 |
|  | Total | 6.9040 | 4.96848 | 59 |

| Source | | Type III Sum of Squares | df | Mean Square | F | Sig. | Partial Eta Squared | Noncent. Parameter |
| --- | --- | --- | --- | --- | --- | --- | --- | --- |
| surface | Sphericity Assumed | 1.029 | 1 | 1.029 | .048 | .827 | .001 | .048 |
|  | Greenhouse-Geisser | 1.029 | 1.000 | 1.029 | .048 | .827 | .001 | .048 |
|  | Huynh-Feldt | 1.029 | 1.000 | 1.029 | .048 | .827 | .001 | .048 |
|  | Lower-bound | 1.029 | 1.000 | 1.029 | .048 | .827 | .001 | .048 |
| surface * group | Sphericity Assumed | 12.505 | 1 | 12.505 | .586 | .447 | .010 | .586 |
|  | Greenhouse-Geisser | 12.505 | 1.000 | 12.505 | .586 | .447 | .010 | .586 |
|  | Huynh-Feldt | 12.505 | 1.000 | 12.505 | .586 | .447 | .010 | .586 |
|  | Lower-bound | 12.505 | 1.000 | 12.505 | .586 | .447 | .010 | .586 |
| Error(surface) | Sphericity Assumed | 1194.103 | 56 | 21.323 |  |  |  |  |
|  | Greenhouse-Geisser | 1194.103 | 56.000 | 21.323 |  |  |  |  |
|  | Huynh-Feldt | 1194.103 | 56.000 | 21.323 |  |  |  |  |
|  | Lower-bound | 1194.103 | 56.000 | 21.323 |  |  |  |  |

| **Tests of Between-Subjects Effects** | | | | | | | | |
| --- | --- | --- | --- | --- | --- | --- | --- | --- |
| Measure: MEASURE_1 | | | | | | | | |
| Transformed Variable: Average | | | | | | | | |
| Source | Type III Sum of Squares | df | Mean Square | F | Sig. | Partial Eta Squared | Noncent. Parameter | Observed Power^a^ |
| Intercept | 96.680 | 1 | 96.680 | 1.382 | .245 | .024 | 1.382 | .211 |
| group | .124 | 1 | .124 | .002 | .967 | .000 | .002 | .050 |
| Error | 3918.694 | 56 | 69.977 |  |  |  |  |  |
| a. Computed using alpha = .05 | | | | | | | | |

| **Descriptive Statistics** | | | | |
| --- | --- | --- | --- | --- |
|  | group | Mean | Std. Deviation | N |
| LevelsiwnGasMP | pronated | 12.9362 | 13.72954 | 30 |
|  | healthy | 16.2279 | 16.18768 | 29 |
|  | Total | 14.5542 | 14.95016 | 59 |
| SandsiwnGasMP | pronated | 11.3797 | 21.08422 | 30 |
|  | healthy | 15.3314 | 16.24525 | 29 |
|  | Total | 13.3220 | 18.80549 | 59 |

| Source | | Type III Sum of Squares | df | Mean Square | F | Sig. | Partial Eta Squared | Noncent. Parameter |
| --- | --- | --- | --- | --- | --- | --- | --- | --- |
| surface | Sphericity Assumed | 2.995 | 1 | 2.995 | .018 | .893 | .000 | .018 |
|  | Greenhouse-Geisser | 2.995 | 1.000 | 2.995 | .018 | .893 | .000 | .018 |
|  | Huynh-Feldt | 2.995 | 1.000 | 2.995 | .018 | .893 | .000 | .018 |
|  | Lower-bound | 2.995 | 1.000 | 2.995 | .018 | .893 | .000 | .018 |
| surface * group | Sphericity Assumed | 10.713 | 1 | 10.713 | .066 | .799 | .001 | .066 |
|  | Greenhouse-Geisser | 10.713 | 1.000 | 10.713 | .066 | .799 | .001 | .066 |
|  | Huynh-Feldt | 10.713 | 1.000 | 10.713 | .066 | .799 | .001 | .066 |
|  | Lower-bound | 10.713 | 1.000 | 10.713 | .066 | .799 | .001 | .066 |
| Error(surface) | Sphericity Assumed | 9124.071 | 56 | 162.930 |  |  |  |  |
|  | Greenhouse-Geisser | 9124.071 | 56.000 | 162.930 |  |  |  |  |
|  | Huynh-Feldt | 9124.071 | 56.000 | 162.930 |  |  |  |  |
|  | Lower-bound | 9124.071 | 56.000 | 162.930 |  |  |  |  |

| **Tests of Between-Subjects Effects** | | | | | | | | |
| --- | --- | --- | --- | --- | --- | --- | --- | --- |
| Measure: MEASURE_1 | | | | | | | | |
| Transformed Variable: Average | | | | | | | | |
| Source | Type III Sum of Squares | df | Mean Square | F | Sig. | Partial Eta Squared | Noncent. Parameter | Observed Power^a^ |
| Intercept | 827.545 | 1 | 827.545 | 2.580 | .114 | .044 | 2.580 | .352 |
| group | 226.539 | 1 | 226.539 | .706 | .404 | .012 | .706 | .131 |
| Error | 17962.727 | 56 | 320.763 |  |  |  |  |  |
| a. Computed using alpha = .05 | | | | | | | | |

| **Descriptive Statistics** | | | | |
| --- | --- | --- | --- | --- |
|  | group | Mean | Std. Deviation | N |
| LevelsiwnGluMP | pronated | 16.7314 | 18.13410 | 30 |
|  | healthy | 18.1763 | 13.78986 | 29 |
|  | Total | 17.4416 | 16.02358 | 59 |
| SandsiwnGluMP | pronated | 11.6261 | 9.79817 | 30 |
|  | healthy | 19.9750 | 17.47796 | 29 |
|  | Total | 15.7298 | 14.60123 | 59 |

| Source | | Type III Sum of Squares | df | Mean Square | F | Sig. | Partial Eta Squared | Noncent. Parameter |
| --- | --- | --- | --- | --- | --- | --- | --- | --- |
| surface | Sphericity Assumed | 215.893 | 1 | 215.893 | 1.402 | .241 | .024 | 1.402 |
|  | Greenhouse-Geisser | 215.893 | 1.000 | 215.893 | 1.402 | .241 | .024 | 1.402 |
|  | Huynh-Feldt | 215.893 | 1.000 | 215.893 | 1.402 | .241 | .024 | 1.402 |
|  | Lower-bound | 215.893 | 1.000 | 215.893 | 1.402 | .241 | .024 | 1.402 |
| surface * group | Sphericity Assumed | 135.056 | 1 | 135.056 | .877 | .353 | .015 | .877 |
|  | Greenhouse-Geisser | 135.056 | 1.000 | 135.056 | .877 | .353 | .015 | .877 |
|  | Huynh-Feldt | 135.056 | 1.000 | 135.056 | .877 | .353 | .015 | .877 |
|  | Lower-bound | 135.056 | 1.000 | 135.056 | .877 | .353 | .015 | .877 |
| Error(surface) | Sphericity Assumed | 8625.804 | 56 | 154.032 |  |  |  |  |
|  | Greenhouse-Geisser | 8625.804 | 56.000 | 154.032 |  |  |  |  |
|  | Huynh-Feldt | 8625.804 | 56.000 | 154.032 |  |  |  |  |
|  | Lower-bound | 8625.804 | 56.000 | 154.032 |  |  |  |  |

| **Tests of Between-Subjects Effects** | | | | | | | | |
| --- | --- | --- | --- | --- | --- | --- | --- | --- |
| Measure: MEASURE_1 | | | | | | | | |
| Transformed Variable: Average | | | | | | | | |
| Source | Type III Sum of Squares | df | Mean Square | F | Sig. | Partial Eta Squared | Noncent. Parameter | Observed Power^a^ |
| Intercept | 62.612 | 1 | 62.612 | .232 | .632 | .004 | .232 | .076 |
| group | 13.829 | 1 | 13.829 | .051 | .822 | .001 | .051 | .056 |
| Error | 15117.704 | 56 | 269.959 |  |  |  |  |  |
| a. Computed using alpha = .05 | | | | | | | | |

| **Descriptive Statistics** | | | | |
| --- | --- | --- | --- | --- |
|  | group | Mean | Std. Deviation | N |
| LevelsiwnRFP | pronated | 17.7739 | 13.39276 | 30 |
|  | healthy | 20.4824 | 16.32180 | 29 |
|  | Total | 19.1052 | 14.83764 | 59 |
| SandsiwnRFP | pronated | 15.3412 | 7.19417 | 30 |
|  | healthy | 19.9442 | 14.24786 | 29 |
|  | Total | 17.6037 | 11.36949 | 59 |

| Source | | Type III Sum of Squares | df | Mean Square | F | Sig. | Partial Eta Squared | Noncent. Parameter |
| --- | --- | --- | --- | --- | --- | --- | --- | --- |
| surface | Sphericity Assumed | 65.431 | 1 | 65.431 | 3.157 | .081 | .053 | 3.157 |
|  | Greenhouse-Geisser | 65.431 | 1.000 | 65.431 | 3.157 | .081 | .053 | 3.157 |
|  | Huynh-Feldt | 65.431 | 1.000 | 65.431 | 3.157 | .081 | .053 | 3.157 |
|  | Lower-bound | 65.431 | 1.000 | 65.431 | 3.157 | .081 | .053 | 3.157 |
| surface * group | Sphericity Assumed | 86.903 | 1 | 86.903 | 4.193 | .045 | .070 | 4.193 |
|  | Greenhouse-Geisser | 86.903 | 1.000 | 86.903 | 4.193 | .045 | .070 | 4.193 |
|  | Huynh-Feldt | 86.903 | 1.000 | 86.903 | 4.193 | .045 | .070 | 4.193 |
|  | Lower-bound | 86.903 | 1.000 | 86.903 | 4.193 | .045 | .070 | 4.193 |
| Error(surface) | Sphericity Assumed | 1160.501 | 56 | 20.723 |  |  |  |  |
|  | Greenhouse-Geisser | 1160.501 | 56.000 | 20.723 |  |  |  |  |
|  | Huynh-Feldt | 1160.501 | 56.000 | 20.723 |  |  |  |  |
|  | Lower-bound | 1160.501 | 56.000 | 20.723 |  |  |  |  |

| **Tests of Between-Subjects Effects** | | | | | | | | |
| --- | --- | --- | --- | --- | --- | --- | --- | --- |
| Measure: MEASURE_1 | | | | | | | | |
| Transformed Variable: Average | | | | | | | | |
| Source | Type III Sum of Squares | df | Mean Square | F | Sig. | Partial Eta Squared | Noncent. Parameter | Observed Power^a^ |
| Intercept | 23.557 | 1 | 23.557 | .086 | .770 | .002 | .086 | .060 |
| group | 39.299 | 1 | 39.299 | .144 | .706 | .003 | .144 | .066 |
| Error | 15307.867 | 56 | 273.355 |  |  |  |  |  |
| a. Computed using alpha = .05 | | | | | | | | |

| **Descriptive Statistics** | | | | |
| --- | --- | --- | --- | --- |
|  | group | Mean | Std. Deviation | N |
| LevelsiwnSTP | pronated | 8.4343 | 14.70400 | 30 |
|  | healthy | 8.5303 | 6.51179 | 29 |
|  | Total | 8.4815 | 11.33917 | 59 |
| SandsiwnSTP | pronated | 5.5562 | 5.73173 | 30 |
|  | healthy | 10.4983 | 7.74649 | 29 |
|  | Total | 7.9854 | 7.18367 | 59 |

| Source | | Type III Sum of Squares | df | Mean Square | F | Sig. | Partial Eta Squared | Noncent. Parameter |
| --- | --- | --- | --- | --- | --- | --- | --- | --- |
| surface | Sphericity Assumed | 22.911 | 1 | 22.911 | .451 | .505 | .008 | .451 |
|  | Greenhouse-Geisser | 22.911 | 1.000 | 22.911 | .451 | .505 | .008 | .451 |
|  | Huynh-Feldt | 22.911 | 1.000 | 22.911 | .451 | .505 | .008 | .451 |
|  | Lower-bound | 22.911 | 1.000 | 22.911 | .451 | .505 | .008 | .451 |
| surface * group | Sphericity Assumed | 102.204 | 1 | 102.204 | 2.010 | .162 | .035 | 2.010 |
|  | Greenhouse-Geisser | 102.204 | 1.000 | 102.204 | 2.010 | .162 | .035 | 2.010 |
|  | Huynh-Feldt | 102.204 | 1.000 | 102.204 | 2.010 | .162 | .035 | 2.010 |
|  | Lower-bound | 102.204 | 1.000 | 102.204 | 2.010 | .162 | .035 | 2.010 |
| Error(surface) | Sphericity Assumed | 2847.431 | 56 | 50.847 |  |  |  |  |
|  | Greenhouse-Geisser | 2847.431 | 56.000 | 50.847 |  |  |  |  |
|  | Huynh-Feldt | 2847.431 | 56.000 | 50.847 |  |  |  |  |
|  | Lower-bound | 2847.431 | 56.000 | 50.847 |  |  |  |  |

| **Tests of Between-Subjects Effects** | | | | | | | | |
| --- | --- | --- | --- | --- | --- | --- | --- | --- |
| Measure: MEASURE_1 | | | | | | | | |
| Transformed Variable: Average | | | | | | | | |
| Source | Type III Sum of Squares | df | Mean Square | F | Sig. | Partial Eta Squared | Noncent. Parameter | Observed Power^a^ |
| Intercept | 536.251 | 1 | 536.251 | 4.164 | .046 | .069 | 4.164 | .518 |
| group | 117.349 | 1 | 117.349 | .911 | .344 | .016 | .911 | .155 |
| Error | 7211.487 | 56 | 128.777 |  |  |  |  |  |
| a. Computed using alpha = .05 | | | | | | | | |

| **Descriptive Statistics** | | | | |
| --- | --- | --- | --- | --- |
|  | group | Mean | Std. Deviation | N |
| LevelsiwnTAP | pronated | 14.7461 | 6.59792 | 30 |
|  | healthy | 20.3178 | 12.63312 | 29 |
|  | Total | 17.4848 | 10.32981 | 59 |
| SandsiwnTAP | pronated | 15.3685 | 7.42252 | 30 |
|  | healthy | 19.9242 | 14.34064 | 29 |
|  | Total | 17.6078 | 11.49367 | 59 |

| Source | | Type III Sum of Squares | df | Mean Square | F | Sig. | Partial Eta Squared | Noncent. Parameter |
| --- | --- | --- | --- | --- | --- | --- | --- | --- |
| surface | Sphericity Assumed | 2.467 | 1 | 2.467 | .096 | .758 | .002 | .096 |
|  | Greenhouse-Geisser | 2.467 | 1.000 | 2.467 | .096 | .758 | .002 | .096 |
|  | Huynh-Feldt | 2.467 | 1.000 | 2.467 | .096 | .758 | .002 | .096 |
|  | Lower-bound | 2.467 | 1.000 | 2.467 | .096 | .758 | .002 | .096 |
| surface * group | Sphericity Assumed | 3.538 | 1 | 3.538 | .137 | .712 | .002 | .137 |
|  | Greenhouse-Geisser | 3.538 | 1.000 | 3.538 | .137 | .712 | .002 | .137 |
|  | Huynh-Feldt | 3.538 | 1.000 | 3.538 | .137 | .712 | .002 | .137 |
|  | Lower-bound | 3.538 | 1.000 | 3.538 | .137 | .712 | .002 | .137 |
| Error(surface) | Sphericity Assumed | 1441.858 | 56 | 25.747 |  |  |  |  |
|  | Greenhouse-Geisser | 1441.858 | 56.000 | 25.747 |  |  |  |  |
|  | Huynh-Feldt | 1441.858 | 56.000 | 25.747 |  |  |  |  |
|  | Lower-bound | 1441.858 | 56.000 | 25.747 |  |  |  |  |

| **Tests of Between-Subjects Effects** | | | | | | | | |
| --- | --- | --- | --- | --- | --- | --- | --- | --- |
| Measure: MEASURE_1 | | | | | | | | |
| Transformed Variable: Average | | | | | | | | |
| Source | Type III Sum of Squares | df | Mean Square | F | Sig. | Partial Eta Squared | Noncent. Parameter | Observed Power^a^ |
| Intercept | 12.787 | 1 | 12.787 | .094 | .761 | .002 | .094 | .060 |
| group | 3.817 | 1 | 3.817 | .028 | .868 | .000 | .028 | .053 |
| Error | 7648.283 | 56 | 136.576 |  |  |  |  |  |
| a. Computed using alpha = .05 | | | | | | | | |

| **Descriptive Statistics** | | | | |
| --- | --- | --- | --- | --- |
|  | group | Mean | Std. Deviation | N |
| LevelsiwnVLP | pronated | 12.8017 | 21.43457 | 30 |
|  | healthy | 10.4781 | 14.97835 | 29 |
|  | Total | 11.6595 | 18.42283 | 59 |
| SandsiwnVLP | pronated | 9.3485 | 16.84030 | 30 |
|  | healthy | 7.3297 | 8.40611 | 29 |
|  | Total | 8.3562 | 13.30215 | 59 |

| Source | | Type III Sum of Squares | df | Mean Square | F | Sig. | Partial Eta Squared | Noncent. Parameter |
| --- | --- | --- | --- | --- | --- | --- | --- | --- |
| surface | Sphericity Assumed | 15.046 | 1 | 15.046 | .291 | .592 | .005 | .291 |
|  | Greenhouse-Geisser | 15.046 | 1.000 | 15.046 | .291 | .592 | .005 | .291 |
|  | Huynh-Feldt | 15.046 | 1.000 | 15.046 | .291 | .592 | .005 | .291 |
|  | Lower-bound | 15.046 | 1.000 | 15.046 | .291 | .592 | .005 | .291 |
| surface * group | Sphericity Assumed | 23.470 | 1 | 23.470 | .453 | .504 | .008 | .453 |
|  | Greenhouse-Geisser | 23.470 | 1.000 | 23.470 | .453 | .504 | .008 | .453 |
|  | Huynh-Feldt | 23.470 | 1.000 | 23.470 | .453 | .504 | .008 | .453 |
|  | Lower-bound | 23.470 | 1.000 | 23.470 | .453 | .504 | .008 | .453 |
| Error(surface) | Sphericity Assumed | 2899.566 | 56 | 51.778 |  |  |  |  |
|  | Greenhouse-Geisser | 2899.566 | 56.000 | 51.778 |  |  |  |  |
|  | Huynh-Feldt | 2899.566 | 56.000 | 51.778 |  |  |  |  |
|  | Lower-bound | 2899.566 | 56.000 | 51.778 |  |  |  |  |

| **Tests of Between-Subjects Effects** | | | | | | | | |
| --- | --- | --- | --- | --- | --- | --- | --- | --- |
| Measure: MEASURE_1 | | | | | | | | |
| Transformed Variable: Average | | | | | | | | |
| Source | Type III Sum of Squares | df | Mean Square | F | Sig. | Partial Eta Squared | Noncent. Parameter | Observed Power^a^ |
| Intercept | 1439.176 | 1 | 1439.176 | 3.764 | .057 | .063 | 3.764 | .479 |
| group | 1755.287 | 1 | 1755.287 | 4.590 | .037 | .076 | 4.590 | .558 |
| Error | 21413.533 | 56 | 382.385 |  |  |  |  |  |
| a. Computed using alpha = .05 | | | | | | | | |

| **Descriptive Statistics** | | | | |
| --- | --- | --- | --- | --- |
|  | group | Mean | Std. Deviation | N |
| LevelsiwnVMP | pronated | 13.3745 | 17.71293 | 30 |
|  | healthy | 18.3958 | 19.07560 | 29 |
|  | Total | 15.8426 | 18.41058 | 59 |
| SandsiwnVMP | pronated | 11.3219 | 15.11338 | 30 |
|  | healthy | 18.1524 | 18.32464 | 29 |
|  | Total | 14.6793 | 16.97573 | 59 |

| Source | | Type III Sum of Squares | df | Mean Square | F | Sig. | Partial Eta Squared | Noncent. Parameter |
| --- | --- | --- | --- | --- | --- | --- | --- | --- |
| surface | Sphericity Assumed | 712.503 | 1 | 712.503 | 5.277 | .025 | .086 | 5.277 |
|  | Greenhouse-Geisser | 712.503 | 1.000 | 712.503 | 5.277 | .025 | .086 | 5.277 |
|  | Huynh-Feldt | 712.503 | 1.000 | 712.503 | 5.277 | .025 | .086 | 5.277 |
|  | Lower-bound | 712.503 | 1.000 | 712.503 | 5.277 | .025 | .086 | 5.277 |
| surface * group | Sphericity Assumed | 294.008 | 1 | 294.008 | 2.177 | .146 | .037 | 2.177 |
|  | Greenhouse-Geisser | 294.008 | 1.000 | 294.008 | 2.177 | .146 | .037 | 2.177 |
|  | Huynh-Feldt | 294.008 | 1.000 | 294.008 | 2.177 | .146 | .037 | 2.177 |
|  | Lower-bound | 294.008 | 1.000 | 294.008 | 2.177 | .146 | .037 | 2.177 |
| Error(surface) | Sphericity Assumed | 7561.189 | 56 | 135.021 |  |  |  |  |
|  | Greenhouse-Geisser | 7561.189 | 56.000 | 135.021 |  |  |  |  |
|  | Huynh-Feldt | 7561.189 | 56.000 | 135.021 |  |  |  |  |
|  | Lower-bound | 7561.189 | 56.000 | 135.021 |  |  |  |  |

| **Tests of Between-Subjects Effects** | | | | | | | | |
| --- | --- | --- | --- | --- | --- | --- | --- | --- |
| Measure: MEASURE_1 | | | | | | | | |
| Transformed Variable: Average | | | | | | | | |
| Source | Type III Sum of Squares | df | Mean Square | F | Sig. | Partial Eta Squared | Noncent. Parameter | Observed Power^a^ |
| Intercept | 47.428 | 1 | 47.428 | .114 | .737 | .002 | .114 | .063 |
| group | 16.675 | 1 | 16.675 | .040 | .842 | .001 | .040 | .054 |
| Error | 23380.832 | 56 | 417.515 |  |  |  |  |  |
| a. Computed using alpha = .05 | | | | | | | | |
